# Engineered combinatorial cell device for wound healing and bone regeneration

**DOI:** 10.3389/fbioe.2023.1168330

**Published:** 2023-05-10

**Authors:** Lucija Kadunc Polajnar, Duško Lainšček, Rok Gašperšič, Petra Sušjan-Leite, Uroš Kovačič, Miha Butinar, Boris Turk, Roman Jerala, Iva Hafner-Bratkovič

**Affiliations:** ^1^ Department of Synthetic Biology and Immunology, National Institute of Chemistry, Ljubljana, Slovenia; ^2^ EN-FIST Centre of Excellence, Ljubljana, Slovenia; ^3^ Department of Oral Medicine and Periodontology, Faculty of Medicine, University of Ljubljana, Ljubljana, Slovenia; ^4^ Institute of Pathophysiology, Faculty of Medicine, University of Ljubljana, Ljubljana, Slovenia; ^5^ Department of Biochemistry, Molecular and Structural Biology, Jozef Stefan Institute, Ljubljana, Slovenia

**Keywords:** cell device, wound healing, bone regeneration, growth factors, engineered cells

## Abstract

Growth factors are the key regulators that promote tissue regeneration and healing processes. While the effects of individual growth factors are well documented, a combination of multiple secreted growth factors underlies stem cell–mediated regeneration. To avoid the potential dangers and labor-intensive individual approach of stem cell therapy while maintaining their regeneration-promoting effects based on multiple secreted growth factors, we engineered a “mix-and-match” combinatorial platform based on a library of cell lines producing growth factors. Treatment with a combination of growth factors secreted by engineered mammalian cells was more efficient than with individual growth factors or even stem cell–conditioned medium in a gap closure assay. Furthermore, we implemented in a mouse model a device for allogenic cell therapy for an *in situ* production of growth factors, where it improved cutaneous wound healing. Augmented bone regeneration was achieved on calvarial bone defects in rats treated with a cell device secreting IGF, FGF, PDGF, TGF-β, and VEGF. In both *in vivo* models, the systemic concentration of secreted factors was negligible, demonstrating the local effect of the regeneration device. Finally, we introduced a genetic switch that enables temporal control over combinations of trophic factors released at different stages of regeneration mimicking the maturation of natural wound healing to improve therapy and prevent scar formation.

## 1 Introduction

Wound healing is sometimes inefficient and may result in scar formation, while the regeneration of the affected tissue could be limited (Maddaluno et al., 2017). Since restoring organ function is crucial for survival, orchestrated and tightly regulated mechanisms that are triggered upon injury enable repair or regeneration of the affected tissue (Devescovi et al., 2008). However, the healing cascade can be disrupted due to infection (Martin and Nunan, 2015), changes from comorbidities, or when the sheer size of the trauma prevents the tissue from restoring its integrity (Lopes et al., 2018), resulting in inadequate healing. Chronic wounds and bone fractures represent a substantial burden for the healthcare system (Burge et al., 2007; Sen et al., 2009), thus there is a pressing need for the development of effective treatments.

Healing of cutaneous wounds and bone defects is a complex process, where an orchestrated interplay of versatile cells, cytokines, and growth factors results in the restoration of tissue integrity and function. Wound healing, in general, comprises four overlapping phases: hemostasis, inflammation, proliferation, and tissue remodeling. Bone healing occurs in similar steps, where soft and hard calluses form after the inflammation phase (Lopes et al., 2018). Stem cells facilitate healing by their differentiation into various cell types; however, recent studies have clearly revealed that stem cells support healing mainly via secretion of several growth factors (GFs) and other molecules rather than via their integration into the tissue (Ferreira et al., 2018). In the wound environment, the most important stem cell–secreted proteins include anti-inflammatory cytokines (Aggarwal and Pittenger, 2009), antimicrobial peptides such as cathelicidins (LL-37) (Krasnodembskaya et al., 2010), and several growth factors that enhance the proliferation and migration of cells and angiogenesis (Ashburner et al., 2000; Chen et al., 2008). On the downside, mesenchymal stem cells (MSCs) have been reported to migrate into various types of tumors, where they could mediate the growth of a tumor and enhance angiogenesis (Motegi and Ishikawa, 2017). Thus, a significant part of the regenerative research focuses on trophic factors. A complex interplay of growth factors and cytokines directs the cellular response to trauma and subsequent tissue repair (Ioannidou, 2006). Growth factors primarily act locally in a paracrine or autocrine fashion and affect a network of cellular processes. For instance, in wound healing, platelet-derived GF (PDGF), fibroblast GF (FGF-2), epidermal GF (EGF), transforming growth factors (TGFs), and others enhance clearance of wounds and also affect cell proliferation and migration to the wound site, thus facilitating healing (Grönberg et al., 2014), while in bone regeneration, bone morphogenetic proteins (BMPs), PDGF, FGF, and vascular endothelial GF (VEGF) enhance migration of osteoprogenitors and together with TGF-β and insulin-like GFs (IGFs) modulate their differentiation and proliferation (Devescovi et al., 2008) Additionally, VEGF and FGF-2 enhance angiogenesis (Grönberg et al., 2014), which is also important in both wound and bone healing (Martin and Nunan, 2015; Lopes et al., 2018). Despite the great potential of GFs, the main obstacle to their widespread use in clinics is their short half-life, in addition to the potential systemic adverse side effects at high doses and high production costs. Additionally, during the successive stages of wound healing, different growth factors are required, which may be difficult to mimic with the administration of purified growth factors.

**FIGURE 1:**
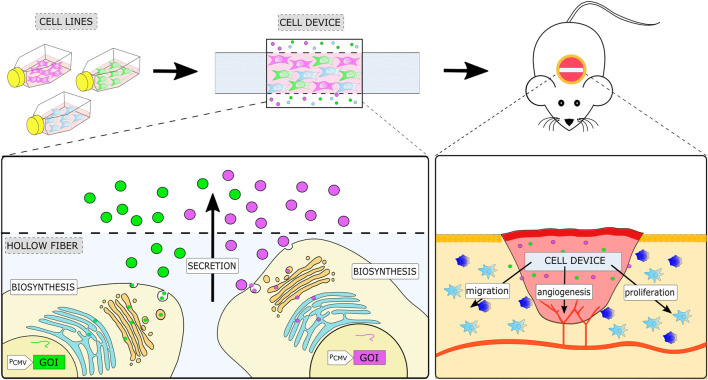
Schematic representation of the therapeutic cell device and its implementation in a mouse model of wound healing. The cell device is prepared by encapsulating the desired combination of engineered stable cell lines, each expressing a single soluble factor into a semipermeable container. A cell device is implanted at the site of injury, where it enhances regeneration processes by local secretion of a combination of growth factors defined by the selected engineered cells.

In this study, we present a cell-based device platform aimed to support regeneration and wound healing that comprises a combination of cells engineered to secrete growth factors and other biologically active proteins. Engineered cells producing growth factors are encapsulated into an inert fiber that protects them from cells of the immune system but enables the exchange of nutrients and the release of active molecules and removal of the device after treatment, thus increasing safety (Figure 1). We first demonstrate that a combination of growth factors is more efficient than single growth factors or stem cell–conditioned medium, and analyze the secretome and effects on target cells. Cell devices are prepared by mixing the desired combinations of cells from the engineered cell line library, which facilitates the adjustment for each therapeutic indication-specific application. We demonstrate the flexibility of this design by producing devices that promote healing in mouse excisional wound and rat calvarial bone defect models. Finally, we present an advanced cell-based device to introduce a variable time course of therapy based on the secretion of different combinations of growth factors during different stages of the regeneration process, mimicking the natural healing process.

## 2 Materials and methods

### 2.1 Construct preparation

All plasmids used were constructed using restriction/ligation or the Gibson assembly method. Murine genes for EGF (NM_010113.3), FGF2 (NM_008006.2), FGF7/KGF (NM_008008.4), HGF (NM_010427.4), IGF1(NM_010512.4), PDGF-B (NM_011057.3), TGF-β1 (NM_011577.1), and VEGFA (NM_001025257.3) were obtained from Sino Biological. Genes for IL-Ra (pORF9-hIL1RNa) and LL-37 (NM_004345.3) were obtained from OriGene. The luciferase reporter gene was PCR amplified from commercial plasmid firefly luciferase from pGL4.16 (Promega). For constitutive expression, soluble factors were amplified using PCR and cloned into the pcDNA3 vector on HindIII/EcoRI or KpnI/NotI site. The Tet-On 3G tetracycline-inducible gene expression systems were used (Clontech). Using the Gibson assembly, a Tet-ON 3G transactivator (pRetroX Tet3G) was cloned downstream of the CMV promoter (pcDNA3 plasmid), and genes of interest (GOI) downstream of the pTRE3GV promoter were inserted into the same plasmid. For the Tet-OFF system, a tetracycline repressor TetR (Smole et al., 2017) fused to the VPR activation domain (Lebar et al., 2019) was used. Using the Gibson assembly TetR-VPR was cloned into pcDNA3.1 downstream of the CMV promotor. In the same plasmid, six repeats of TRE binding sites following minimal promoter controlling GOI were cloned.

### 2.2 Cell cultures

The NIH-3T3 cell line (ECACC) was cultured in the DMEM F-12 medium (Invitrogen) supplemented with 5% fetal bovine serum (Gibco) at 37°C in a 5% CO_2_ environment.

Mouse skin fibroblasts (MSFs) were isolated as described in Lichti et al. (2008). Briefly, the skin was removed from newborn C57BL/6 mice and incubated floating on trypsin. The dermis was separated from the epidermis and cut into 1 mm pieces. The dermis was digested using collagenase (Roche, 2.5 mg/mL) and after a series of centrifugations, the pellet was filtered (20 mm mesh, Falcon). The cells were seeded in DMEM +10% FBS and incubated for an hour.

The protocol for isolation of ADSC was adapted from Moreno-Fernandez et al. (2021) and Araña et al. (2013). Mouse adipose tissue from C57BL/6 mice was processed using gentleMACS Dissociator (Miltenyi Biotec) using a user-defined gentleMACS Program. Afterward, collagenase was added, and the tissue was incubated for an hour at 37°C, in 5% CO_2_, under continuous rotation using the MACSmix Tube Rotator (Miltenyi Biotec). Following incubation, normal culture medium (DMEM supplemented with 10% FBS) was added, and the cells were centrifuged at 420 *g* for 10 min. The adipose tissue and medium were discarded, and the pellet was resuspended in 10 mL of normal medium and seeded into a T75 flask. The cells were sorted using the MACS magnetic sorter (Miltenyi Biotech) and mouse CD105 MultiSort Kit (Miltenyi Biotech) according to the manufacturer’s instructions.

### 2.3 Stem cell differentiation and characterization

Isolated SCs were seeded into a 12-well plate at 1 × 10^4^ cells/cm^2^ and cultured in DMEM supplemented with 10% FBS. After 7 days in culture, the medium was replaced with osteoblast (StemPro^®^ Osteogenesis Differentiation Kit, Gibco) or adipocyte differentiation media (StemPro^®^ Adipogenesis Differentiation Kit, Gibco); controls were kept in DMEM supplemented with 10% FBS. The medium was exchanged every 2–3 days for 7 days (adipocyte) or 24 days (osteoblast) of differentiation. Afterward, the differentiation was assessed via specific staining. For adipocyte differentiation, Oil Red O stain was used. The osteoblast differentiation was confirmed using Alizarin Red S stain and measuring by alkaline phosphatase.

### 2.4 Stable cell line preparation and characterization

The NIH-3T3 cells were seeded in a 10-cm Petri dish. At 80% confluence, they were transfected with a combination of DNA and PEI (6 μL PEI/500 ng DNA, PEI stock concentration 0.324 mg/mL, pH 7.5). Two days post transfections, the cells were selected and maintained in medium containing G418 (1.2 mg/mL; Sigma). To confirm the secretion of soluble factors, each cell line was cultured in a Petri dish in a serum-free DMEM F-12 medium. For inducible systems, doxycycline (1 μg/mL) was added. Then, 24 h after seeding, the supernatants were collected and concentrations of secreted soluble factors were determined using qPCR (KGF and LL-37) or an appropriate ELISA kit [EGF, FGF-2, IGF-I, HGF, VEGF-A (Abcam); PDGF-B (R&D Systems); TGF-β1, IL1-Ra (eBioscience)] according to the manufacturer’s instructions. Reporter cell lines (fLuc ON/OFF) were characterized by measuring luciferase expression. The inducibility of the cells was tested by adding doxycycline in concentrations 0.01–10 μg/mL.

### 2.5 qPCR

Stable cell lines expressing KGF or LL-37 (OFF system) and control untransfected NIH-3T3 cells were seeded into 6-well plates. One plate of LL-37 OFF was cultured with doxycycline (1 μg/mL) and another without doxycycline. Upon reaching 80% confluence, RNA was isolated using a PureLink RNA Mini Kit (Thermo Fischer Scientific). cDNA synthesis was performed using 1 µg of total RNA using a High-Capacity cDNA Reverse Transcription Kit (Thermo Fisher Scientific). The real-time quantitative PCR (qPCR) assays were performed using Light Cycler 480 (Roche). To evaluate gene expression, the following primers were used: *kgf* (forward 5′ AAG GGA CCC AGG AGA TGA AGA ACA G 3′, reverse 5′ GTA CGA ATT CTT AGG TTA TTG CCA TAG GAA GAA AAT GG 3′), *LL37* (forward 5′ GGT GAA GCG GTG TAT GGG GAC AGT GAC CC 3′ reverse 5′ ATG GAT ATC TGC AGA ATT CCC TAG GAC TCT GTC CTG GGT AC 3′), and *gapdh* (forward 5′ TTC ACC ACC ATG GAG AAG GC 3′, reverse 5′ GGC ATG GAC TGT GGT CAT GA 3′). The relative transcription levels were calculated using the Ct (2^−ΔΔCT^) method.

### 2.6 Gap closure assay

MSF cells were seeded into a cell insert (ibidi) inserted into a 24-well plate at a density of 7 × 10^5^ cells/mL and cultured for a day to reach confluence. Conditioned media were prepared by culturing untransfected cells (control) and each cell line in a Petri dish in serum-free DMEM F-12 medium. After 2 days, the media were collected and concentrations of secreted soluble factors were determined using an appropriate ELISA kit. Afterward, culture inserts were removed, and conditioned media (80 µL) mixed with fresh serum-free DMEM F-12 medium (50 µL) were added. For testing the effect of a single GF, conditioned medium was prepared by using 10 µL of used medium (by single stable cell line) and mixing it with 70 µL of used medium from control cells (NIH-3T3 that are not secreting GF). Conditioned medium for testing the MIX effect was prepared by mixing 10 µL of each stable cell line medium to get 80 µL of the mixture. After 24 h, cell gap closure was observed, and images were captured using a Leica DM IL LED inverted microscope with a mounted camera Leica MC170 HD. The images were analyzed and quantified using the program ImageJ (1.52a) with the extension MRI Wound Healing Tool.

### 2.7 Metabolic activity

MSF cells were seeded into a 96-well plate, and after a day in culture, conditioned medium was added. The cells were incubated for 24 h. Afterward, an XTT reagent (10 mM, Sigma) was added to the cells to determine cell metabolic activity. The cells were incubated for 4 h and then absorbance was measured at 490 nm and 680 nm. The absorbance was calculated as A_490_-A_680_.

### 2.8 RNA isolation and sequencing

Primary MSF cells were seeded into a 6-well plate and upon reaching 80% confluence, condition media (control, GF combination, or SC) were added. After 24 h, RNA was isolated using a PureLink RNA Mini Kit (Thermo Fischer Scientific), and a single replicate per condition was sent to sequencing (Arraystar Inc.). The mRNA was enriched by oligo (dT) magnetic beads. An Illumina kit for the RNA-seq library was used to prepare RNA-seq libraries. Libraries were qualified using Agilent 2100 Bioanalyzer for concentration, fragment size distribution (400–600 bp), and adapter dimer contamination. The amount was determined by the absolute quantification qPCR method. The barcoded libraries were mixed in equal amounts and used for sequencing on the instrument. Sequencing was carried out using the Illumina HiSeq 4000 according to the manufacturer’s instructions by running 150 cycles. Afterward, fragments were 5′, 3′-adaptor trimmed and filtered *≤* 20 bp reads with the Cutadapt software. The trimmed reads were aligned to reference genome with the HISAT 2 software. Differentially expressed gene analyses were performed with R package ballgown. Fold change (cutoff 1.5), *p*-value (≤0.05), and FPKM (≥0.5 mean in one group) were used for filtering differentially expressed genes. For gene enrichment analysis, we used enrichment analysis tool g:Profiler (Raudvere et al., 2019) with a user threshold of 0.05 and selected GO:BP (The Gene Ontology Consortium, 2018) and Reactome (Jassal et al., 2020). For visualization of the enriched data, we used the Cytoscape 3.8.0 (Shannon et al., 2003) program and its apps EnrichmentMap (Merico et al., 2010) and AutoAnnotate (*p*-value: 1.0; FDR Q-value: 0.01; Jaccard: 0.25; test: Jaccard index). Clusters of related genes were automatically annotated and manually corrected.

### 2.9 Secretome analysis

Non-transfected NIH-3T3 cells, a combination of stable cell lines (EGF, FGF-2, HGF, IGF-I, KGF, PDGF-B, TGF-β1, VEGF-A) and stem cells, were seeded into a 6-well plate (1 × 10^6^ cells/well). The next day the media were changed to DMEM F12 without FBS. After 24 h, the media were collected (triplet for each condition) and sent for Shotgun LC-MS/MS analysis (VIB-UGent Center for Medical Biotechnology).

Peptides (2 µg) were injected into the Thermo Scientific™ UltiMate™ 3000 RSLCnano system in-line connected to an Orbitrap Fusion Lumos mass spectrometer (Thermo). Trapping was performed in solvent A [0.1% trifluoroacetic acid in water/acetonitrile (ACN) (98:2, v/v)] at 10 μL/min for 4 min on a 20-mm trapping column (made in-house, 100 μm internal diameter (I.D.), 5 μm beads, C18 ReproSil-HD, Dr. Maisch, Germany). 200 cm µPAC™ column (C18-endcapped functionality, 300 µm wide channels, 5 µm porous-shell pillars, inter pillar distance of 2.5 µm, and a depth of 20 μm; PharmaFluidics, Belgium) was used to separate the peptides. The mass spectrometer was automatically switching between MS and MS/MS acquisition. Full-scan MS spectra (300–1500 m/z) were acquired in 3 s acquisition cycles at a resolution of 120,000 in the Orbitrap analyzer after accumulation to a target AGC value of 200,000 with a maximum injection time of 250 ms. The fragments were analyzed in the Ion Trap Analyzer at a normal scan rate. For data analysis, MaxQuant (version 1.6.9.0) using the Andromeda search engine with default search settings that included a false discovery rate set at 1% at both the peptide and protein levels was used, and spectra were searched against the mouse proteins in the SWISS-PROT database (www.uniprot.org). The Perseus software (version 1.6.2.1) was used for further data analysis. The results were quantified using a *t*-test (FDR = 0.05). For the identification of unique proteins, the BioVenn application was used (Hulsen et al., 2008). For protein enrichment analysis, we used enrichment analysis tool g:Profiler (Raudvere et al., 2019) with a user threshold of 0.05 and selected GO:BP (The Gene Ontology Consortium, 2018) and Reactome (Jassal et al., 2020). For visualization of enriched data, we used the Cytoscape 3.8.0 (Shannon et al., 2003) program and its apps EnrichmentMap (Merico et al., 2010) and AutoAnnotate (*p*-value: 1.0; FDR Q-value: 0.01; Jaccard: 0.25; test: Jaccard index). Clusters of related proteins were automatically annotated and manually corrected.

### 2.10 Device preparation and validation

For the preparation of the cell device, polyvinylidene fluoride (PVDF) hollow fibers KrosFlo Implant Membranes with 500 kDa (20 nm) pores (Spectrum Labs) were used. Encapsulation of cells was performed as described by Zhang and Kaelin (2005). Briefly, the hollow fiber was filled with a stable cell line or combination of cell lines (4.2 10^6^ cell/mL, in the case of combination, cell lines were mixed at equal ratios) and heat-sealed in 1–1.2 cm long segments. The sealed fibers were cultivated in a 6-well plate. To confirm the long-term production and secretion of GF/SF from the cell device, it was cultured for up to 25 days. The media were changed daily, and the supernatants were analyzed using ELISA. Secretion of the GF from the cell device was confirmed using the supernatants. To confirm the switching ability of the device *in vitro*, cell lines stably expressing OFF or ON systems were encapsulated and cultured in 12-well plates. Hollow fibers were visualized before and 24 h after doxycycline induction (1 μg/mL) using IVIS^®^ Lumina Series III (Perkin Elmer). The data were analyzed with Living Image^®^ 4.5.2 (Perkin Elmer).

### 2.11 Flow cytometry

A flow cytometry analysis was performed using a flow cytometer CyFlow (Partec). The cells were washed with FACS buffer (PBS, 2% FBS) and resuspended in a 0.1 mL FACS buffer. The cells were stained with anti-mouse CD105-APC, CD90.2-FITC, CD34-FITC, CD11b-APC (Miltenyi Biotec), CD14 PerCP‐Cy5.5 (eBioscience), Ly-6A/E (Sca-1)-Pacific Blue (BioLegend) antibodies. Data were analyzed using the FlowJo software (BD).

### 2.12 Animal experiments

All animal experiments were performed according to the directives of the EU 2010/63 and 3R principles and were approved by the Administration of the Republic of Slovenia for Food Safety, Veterinary, and Plant Protection of the Ministry of Agriculture, Forestry, and Foods, Republic of Slovenia (Permit Number U34401-55/2013/23, U34401-23/2015/8).

For excisional wound healing, C57Bl/6J-OlaHsd mice were purchased from Harlan (Italy). For the TET-inducible system experiments, Balb/c mice (Harlan, Italy) were used. Eight- to twelve-week-old male and female mice with normal health and immune status were included in the experiments. The animals were randomly assigned to experimental groups. No power calculations were performed to choose the group size. The mice were fed with standard chow and tap water *ad libitum*. They were housed in IVC cages at a regulated 12-h dark/light cycle regime.

For the bone regeneration model and evaluation of cell survival, male Wistar rats were used. They were contained at room temperature at a maximum of three rats in a single cage and offered standard chow and water *ad libitum*.

### 2.13 Mouse model of excisional wound healing

The mice were sedated using xylazine (10 mg/kg; Chanazine; Chanelle) and ketamine (100 mg/kg; Bioketan; Vetoquinol Polska) combination, which was administered intraperitoneally. If necessary, anesthesia was also maintained with 1.4%–2% of inhaled isoflurane (Forane; Arkema) at a flow rate of 1.5 L/min (Harvard Apparatus). All mice received post-operative analgesia meloxicam (1–2 mg/kg/12 h; Metacam; Vetmedica). Mouse back was shaved, and an appropriate surgical field was prepared by applying multiple rounds of 70% ethanol and povidone-iodine. An excisional round-shaped wound was produced by using a disposable 10 mm Biopsy punch scalpel (Kai Medical). Afterward, a 12-mm silicone wound splint (Grace Bio-Labs) was placed around the wound and sutured to the skin using 5/0 non-absorbable sutures Silkam (B.Braun). In the middle of the wound, the cell device was placed and fixed to the wound by using 5/0 non-absorbable sutures Silkam (B.Braun). The wound was covered with a transparent film dressing Tegaderm™ Film (3M Medical). In some control animals, the silicone wound splint was attached to the skin of the animals at the end of the experiments with only two sutures so that they were removed when taking pictures, but they were present for the whole experiment to avoid skin contraction during wound healing.

After the defined period, the mice were humanely sacrificed with CO_2_ and exsanguination, and the wound samples were collected for further analysis. In experiments with cell devices, containing TET ON/OFF systems, all mice (control and device) received doxycycline (20 μg/g) intraperitoneally 48 h, 72 h, and 144 h after the excisional wound procedure.

### 2.14 Mouse model for TET-inducible system monitoring

To determine the functionality of the cell device, containing the TET-ON/OFF-fLuc system, implant devices were placed subcutaneously. Briefly, BALB/c mice were anesthetized with isoflurane (Forane; Arkema) inhalation (approximately 2% at a flow rate of 1.5 L/min). After the surgical field was prepared, a simple 5 mm surgical wound was made at the back of the mice. The cell device was implanted subcutaneously. The wound was closed with a simple 5/0 suture. The mice received doxycycline (20 μg/g, Sigma) intraperitoneally. After 24 h, the mice were anesthetized with isoflurane. Each mouse received 150 mg/kg of body weight of D-luciferin (Xenogen) intraperitoneally. After 10–15 min, the mice were *in vivo* imaged using IVIS^®^ Lumina Series III (Perkin Elmer). The data were analyzed using Living Image^®^ 4.5.2 (Perkin Elmer).

### 2.15 Cell survival *in vivo*

To determine the survival of cells *in vivo*, a cell device was implanted subcutaneously above the limb. Briefly, the rats were anesthetized with an intraperitoneal injection of 2% xylazine (Chanazine, Chanelle 8 mg/kg) and ketamine chloride (Bioketan, Vetoquinol Polska, 60 mg/kg). After the surgical field was prepared, a simple surgical wound was made above the limb, and the cell device was implanted subcutaneously. After 4 weeks, the device was removed and placed on a 12-well plate with 1 mL of DMEM F12 + 5% FBS for 3 days. Afterward, the medium was collected, and the concentration of TGF-β1 was measured using ELISA.

### 2.16 Rat model of bone regeneration

All calvarial defect model experiments were created in deep anesthesia with an intraperitoneal injection of 2% xylazine (Chanazine, Chanelle 8 mg/kg) and ketamine chloride (Bioketan, Vetoquinol Polska, 60 mg/kg). A midline incision through the skin that was reflected using a surgical retractor, allowed access to the calvarial bone. After the periosteum was cut and retracted using blunt dissection, the calvarial bone surfaces were exposed. Using a standardized sterile trepanation bur, two calvarial bone defects with a diameter of 3 mm were created in each animal under copious irrigation with room temperature saline. The bur was inserted into a handpiece and connected to an electric motor with a speed of up to 14.000 spins per minute (Iskra, Slovenia). The midsagittal sutures were not included in the bone defects to eliminate any effects that they might have on bone healing. The cell devices were placed directly above both defects and fixed to the nearby periosteum with resorbable sutures. The periosteum layer above the defect was sutured by absorbable sutures. Two months after the establishment of calvarial defects, the animals were euthanized by CO_2_ and exsanguination. The parietal bone was harvested and stored in 10% buffered formaldehyde for further analysis. Combo5 cell devices used in the experiment were collected. The cells were flushed out of hollow fibers, and their viability was determined using trypan blue and cell counter Countess™ (Invitrogen).

### 2.17 Micro-CT scanning

Scans of excised rat skulls were performed using a Quantum Fx micro-CT scanner (PerkinElmer) in 50 mL Falcon tubes filled with PBS solution to assess the bone regeneration of 3-mm drilled holes in the skull before it was placed into decalcification solution. The micro-CT scanner operated at 90 kV voltage and 160 µA electric current. Standard quality scans were taken for positioning and high-quality scans for 3D reconstruction at magnifications: FOV 20 mm, 17 s and 4.5 min with a pixel size of 40 μm and FOV 10 mm, 26 s and 3 min with a pixel size of 20 μm, respectively. Additionally, two separate scans were taken for each hole separately at FOV 5 mm, 26 s and 3 min with a pixel size of 10 µm. To evaluate bone regeneration, 3D images were created using the free 3D Slicer software and Quantum Fx micro-CT viewer V.1.3.0 software (PerkinElmer). The surface area was determined using the ImageJ (1.52a) program.

### 2.18 Histology

Wound samples of mice that underwent excisional wound healing procedures were fixed overnight in 10% neutral buffered formalin (Sigma Aldrich) and then embedded in paraffin (Leica Paraplast). The paraffin blocks were cut 7 μm thick with a rotation microtome RM 2245 (Leica). Tissue sections underwent deparaffinization and rehydration using xylene and different dilutions of ethanol (Sigma Aldrich). Tissue slides were then stained with hematoxylin and eosin (H&E) and Mason’s trichrome stain and then visualized using Leica DMi8 with a Leica MC170 HD camera. Calvarial specimens of rats were fixed in 10% buffered formalin (pH = 7.4). The bone was decalcified in 10% ethylenediaminetetraacetic acid (EDTA) at room temperature for 30 days. The specimens were then dehydrated in progressive solutions of ethanol (70, 90, and 100%), cleared in xylol, and embedded in paraffin before sectioning to the 9 µm thickness using a rotatory microtome (Leica RM 2135, Leica Microsystems, Nussloch, Germany). Sections were stained with H&E. A qualitative analysis of the histologic samples was done on a light microscope (Zeiss-Opton, Germany). Before the analysis, we viewed the complete series of sections of each particular specimen and selected the ones displaying the widest dimension of the unhealed defect.

## 3 Results

### 3.1 Combination of growth factors is superior to stem cell–conditioned medium in accelerating *in vitro* wound healing

To test the effect of combinations of growth factors on cutaneous wound healing, several growth factors were selected for the preparation of cell lines. EGF, FGF-2, HGF, IGF-I, KGF, and TGF-β1 have been previously shown to enhance reepithelization and fibroblast migration (Singer and Clark, 1999; Martin and Nunan, 2015). FGF-2 and VEGF-A enhance angiogenesis and consequently tissue oxygenation and thus contribute to one of the key processes of wound healing (Bodnar, 2015). TGF-β1, EGF, FGF-2, and HGF also induce the epithelial–mesenchymal transition of keratinocytes, thus enabling wound reepithelization (Haensel and Dai, 2018). We prepared a library of stable cell lines based on mouse fibroblast cell line NIH-3T3, where a single growth factor gene was integrated into the genome of each newly established cell line. Secretion of each growth factor into the cell medium was confirmed by ELISA or qPCR (Supplementary Figure S1). We compared the functionality of this multi-cell line platform with stem cell–based secretome. First, we isolated adipose-derived stem cells (SCs). The isolated SCs were adherent, expanding in culture and expressing appropriate surface markers of stemness (Supplementary Figures S2A, B). The differentiation potential of the isolated SCs was confirmed by inducing osteogenic and adipogenic differentiation (Supplementary Figures S2C–E).

**FIGURE 2:**
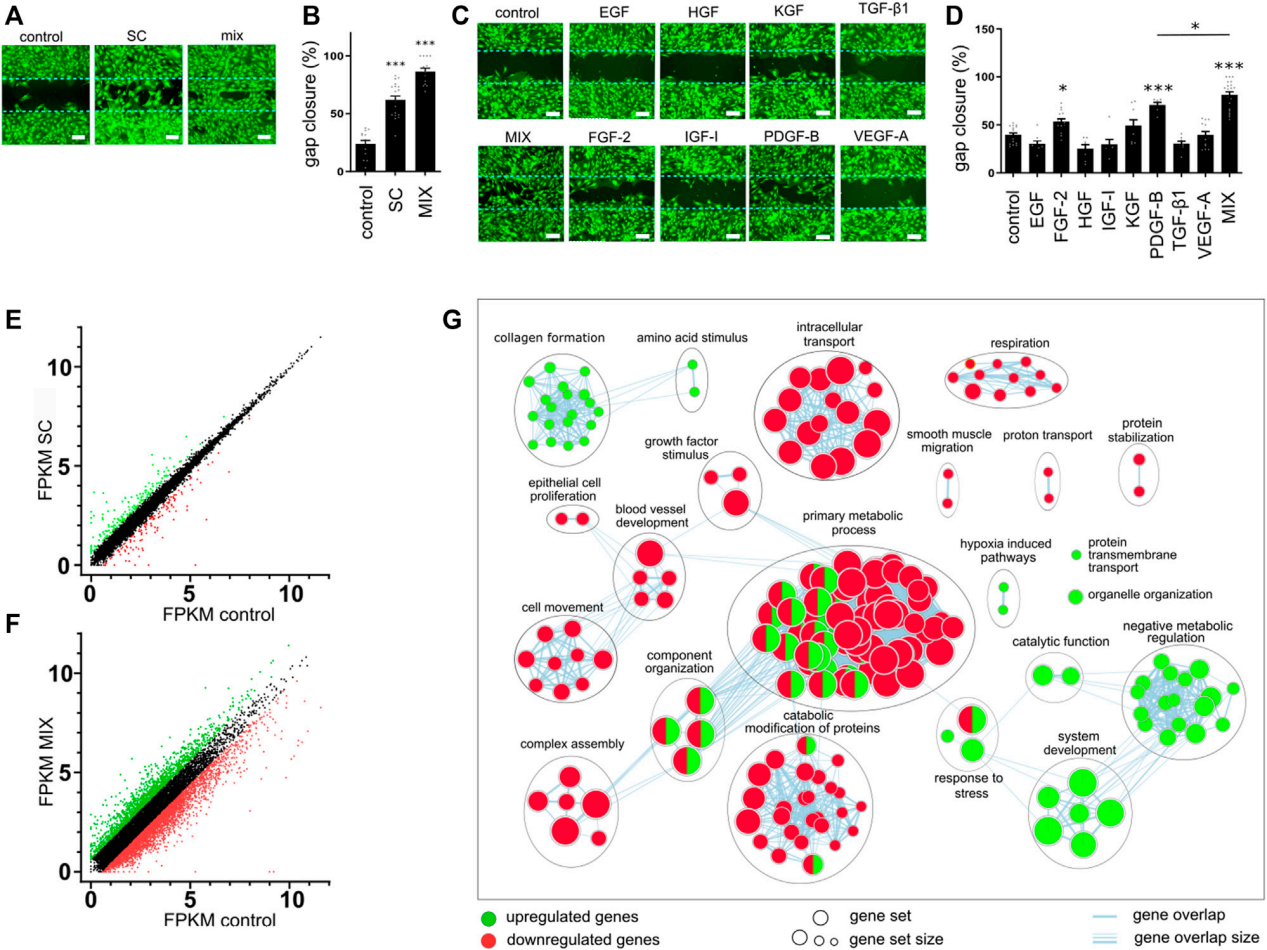
Effect of conditioned media on gap closure and gene expression. **(A–D)** Mouse skin fibroblast (MSF) cell migration was evaluated using a gap closure assay. Equal numbers of primary MSFs were seeded into wells with ibidi culture insert. After 24 h, inserts were removed and cells were treated with conditioned media containing a single growth factor or their combination (EGF, 15 pg/mL; FGF-2, 80 pg/mL; HGF, 700 pg/mL; IGF-I, 25 pg/mL; KGF, PDGF-B, 400 pg/mL; TGF-β, 1130 pg/mL; and VEGF-A, 100 pg/mL), control medium, or stem cells medium. Migration was assessed after 24 h. **(A,C)** Cells were stained with calcein AM and visualized using fluorescent microscopy. The bar represents 200 μm. **(B,D)** Images obtained with fluorescent microscopy were quantified, and gap closure was calculated for each sample. The results in **(A)** and **(C)** are representative images of at least three independent experiments. The mean ± SEM of at least seven replicates from three independent experiments are presented in **(B)** and **(D)**. Statistical analysis with one-way ANOVA (*p < 0.05; **p < 0.01; ***p < 0.001). **(E–G)** MSF cells were seeded into a 6-well plate and media conditioned with control cells, stem cells, or the combination of cells secreting growth factors were added. After 24 h, RNA was isolated and sequenced. Differentially expressed genes in samples of SC **(E)** or combination **(F)** when compared to the control were identified. **(G)** Enrichment map of selected annotated categories involved in wound healing of up- and downregulated genes in MSF cells treated with a combination of GF when compared to control.

To determine the effect of conditioned media on cell migration, we performed a gap closure assay. Mouse primary skin fibroblasts (MSFs) were used as a relevant biological model. Since serum contains a diversity of growth factors and other molecules that could accelerate wound healing, serum-free conditioned medium was used to test the effect of SCs and single or combinations of produced growth factors. In line with the previous report (Park et al., 2018), SC-conditioned medium improved cell migration and gap closure in fibroblasts (Figures 2A, B). While the addition of FGF-2- and PDGF-B-conditioned media enhanced cell migration when compared to the control cell medium, the combination of eight growth factors (EGF, FGF-2, HGF, IGF-I, KGF, PDGF-B, TGF-β, and VEGF-A), MIX, promoted gap closure much better than any single growth factor (Figures 2C, D). However, the combination of GFs (MIX) exhibited an even better effect on wound closure than SC (Figures 2A, B). No single factor except PDGF-B and TGF-β1 affected the metabolic activity of cells (Supplementary Figure S3). In the presence of the SC-conditioned medium, a decrease in metabolic activity was observed, whereas the combination of GFs caused an increase in proliferation (metabolic activity) (Supplementary Figure S3).

To further investigate and compare the cellular response to the addition of conditioned media containing combinations of growth factors, we performed the transcriptome analyses of MSF cells treated with either SC- or MIX-conditioned media for 24 h, corresponding to the time point when the gap closure efficiency was assessed. As a control, we selected MSF cells treated with NIH-3T3-conditioned medium and compared their gene expression to MSF cells treated with a combination of conditioned medium (MIX) or SC-conditioned medium. In SC-treated MSF cells, there were 162 upregulated and 128 downregulated genes when compared to the control (Figure 2E), while in MIX-conditioned MSF cells, there were 1,560 upregulated and 3,159 downregulated genes (Figure 2F). To gain an insight into the cellular response to different treatments, we performed a gene set enrichment analysis with g:Profiler (Raudvere et al., 2019) using the Gene Ontology (GO) (Ashburner et al., 2000; The Gene Ontology Consortium, 2018) and Reactome database (Jassal et al., 2020). There were no enriched pathways in upregulated pathways and only nine enriched downregulated pathways in the SC-conditioned samples (Supplementary Figure S4A). The weak effect of the SC-conditioned medium might be due to the low concentration of secreted GFs. On the other hand, in cells conditioned with MIX, there were 123 (upregulated) and 257 (downregulated) enriched pathways (Supplementary Figures S4B, C). The network enrichment map of MIX-conditioned MSF using Cytoscape (Reimand et al., 2019; Shannon et al., 2003) shows upregulation in negative metabolic function and system development (Figure 2G). As fibroblasts support wound healing, particularly through extracellular matrix formation and collagen deposition, upregulation of the pathways connected to collagen formation and extracellular matrix organization suggests that the MIX had positive effects on fibroblasts *in vitro* and that pathways that lead to improved wound healing were engaged (Xue and Jackson, 2015). Hypoxia-induced pathways and response to stress were increased, correlating with an enhanced rate of cell migration *in vitro*. Growth factors activate multiple signaling pathways to affect cell survival, proliferation, growth, and other processes (Gross and Rotwein, 2016) and coordinate and control metabolism to allow the proliferation of cells to satisfy the energy demands (Mason and Rathmell, 2011). Some biological categories were downregulated, such as growth factor stimulation, which could be explained by the activation of growth factor negative feedback loops at prolonged stimulation times, such as reported in the case of EGF (Amit et al., 2007).

**FIGURE 3:**
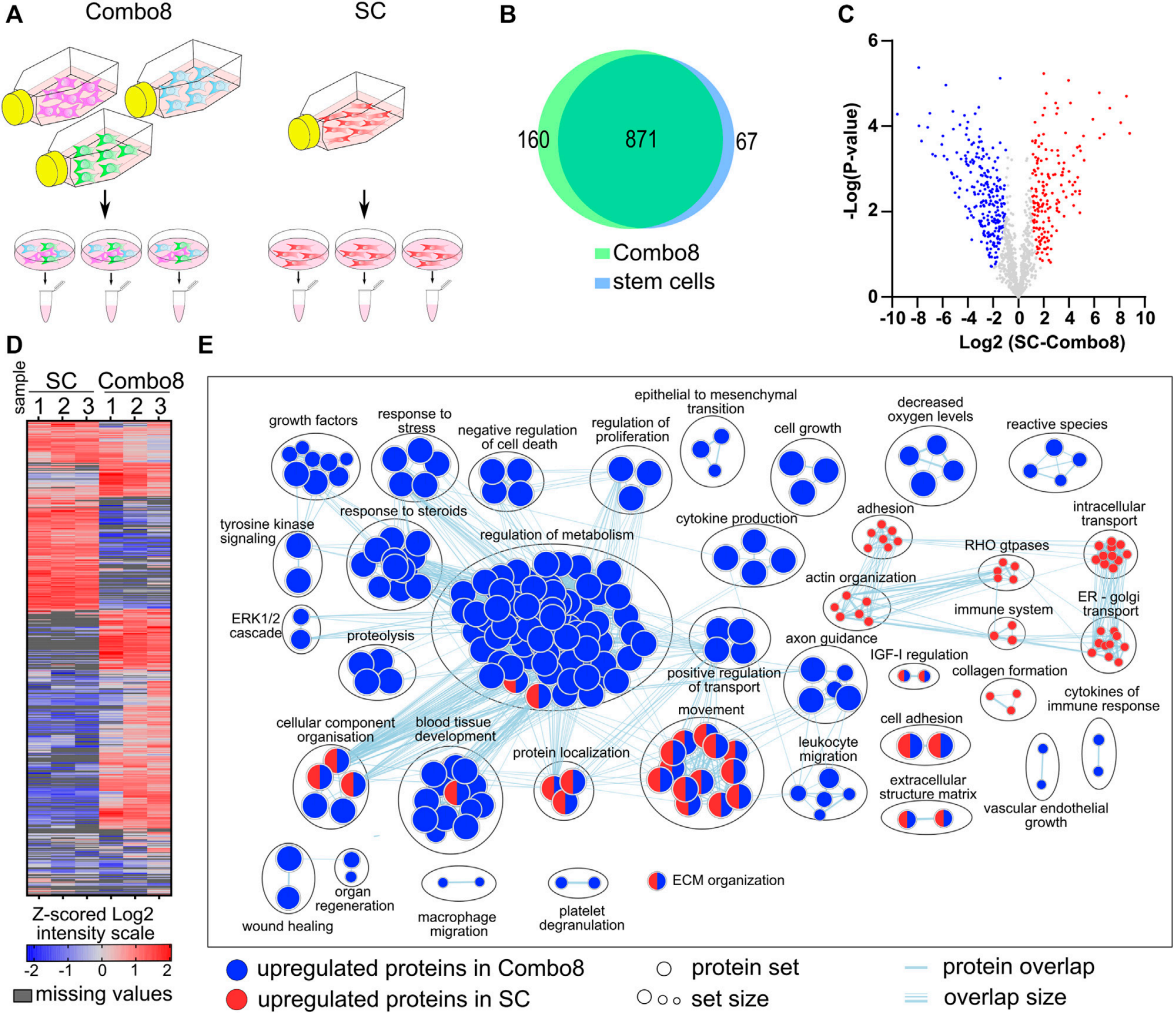
Analysis and characterization of the secretome of stem cells when compared to the combination of stable cells secreting eight GFs. **(A)** Workflow of sample preparation for LC-MS/MS analysis. **(B)** Venn diagram of proteins identified in samples of stem cells and Combo 8. **(C)** Volcano plot of significantly upregulated proteins in Combo 8 (blue) and in SC (red). Statistical analysis, *t*-test (FDR = 0.05, S_0_ = 1). **(D)** Non-supervised hierarchical clustering of proteins with significantly changed abundance (*t*-test, FDR = 0.05, S_0_ = 1). Expression values log_2_ were Z-score–normalized for each sample. Columns represent samples and rows represent individual proteins (blue, low expression; red, high expression; grey, missing values). **(E)** Enrichment map with wound healing–related annotated categories of upregulated proteins in Combo 8 (blue) and stem cells (red).

To better understand the effect of conditioned media on cell proliferation and gene expression, we additionally analyzed the secretome of the control, SC, and combination of cells secreting eight factors (Combo 8) (Figure 3A). The secretome analysis revealed 1,117 different proteins in the samples of the control, SCs, and Combo 8, of which 822 proteins were found in all three samples. A comparison of SCs or Combo 8 with the control revealed important differences in protein abundance (Supplementary Figure S5). When comparing the SC secretome to the Combo 8 secretome, 871 proteins were identified in both samples, 67 unique proteins were found in the SC secretome, and 160 unique proteins were found in the Combo 8 secretome (Figure 3B). When comparing protein abundance, 170 upregulated proteins were identified in the SC secretome and 249 upregulated proteins in the Combo 8 samples (Figures 3C, D). To uncover differences in the secretome profile, an enrichment analysis was performed of differentially expressed proteins in the SC or Combo 8 samples compared to the control (Supplementary Figures S5A–D) or SCs compared to the Combo 8 samples (Figure 3E) using g:Profiler (Raudvere et al., 2019) and the results visualized using Cytoscape (Shannon et al., 2003) with an app enrichment map (Merico et al., 2010). In the SC samples, enriched proteins that are involved in the cytoskeletal organization and intracellular transport (Abreu-Blanco et al., 2012) were identified. Although we used cell lines overexpressing eight growth factors (EGF, FGF-2, HGF, IGF-I, KGF, PDGF-B, TGF-β1, and VEGF-A), we observed substantial differences in the levels of other proteins as well. We identified numerous biological categories associated with enhanced healing. Epithelial to mesenchymal transition enhances keratinocyte migration and consequently re-epithelization (Haensel and Dai, 2018), and immune cells that migrate to the wound site eliminate pathogens and secrete growth factors and cytokines (Ellis et al., 2018). Both cytokine and different growth factor categories were also enriched in the upregulated protein set. GFs signal predominately via the ERK1/2 pathway (Mebratu and Tesfaigzi, 2009), which was upregulated due to overexpression of GFs in the MIX. Taken together, the secretome analysis of a combination of cells producing growth factors revealed many additional upregulated proteins that are known to directly enhance healing (Figure 3E), confirming the therapeutic potential of our designed cell lines. The *in vitro* wound healing model demonstrated that the medium of a combination of cells that were engineered to release the eight GFs improved wound healing when compared to a single growth factor secreting cells and stem cells through the enhancement of multiple pathways connected to wound healing.

### 3.2 Engineered cell device comprising cell combination for secretion of growth factors promotes wound healing in a mouse model

After confirming the secretion and biological activity of GFs produced by the combination of designed cell lines *in vitro*, we set out to prepare the cellular device by encapsulating cells in hollow polyvinylidene fluoride (PVDF) fiber with 20-nm pores (Figure 1). The goal was to prepare a platform that could be suited to the desired therapeutic application by a selection of cells secreting the desired GFs. Encapsulation protects GF-secreting cells from the host's immune system but permits the free exchange of nutrients and secreted therapeutic proteins. Encapsulation also enables the local containment of engineered cells, increasing the safety of our approach. We indeed confirmed that GFs are secreted from the cell device for at least 14 days (Supplementary Figures S6A–D). To evaluate cell survival and long-term production of growth factors *in vivo*, the cell device secreting TGF-β1 was implanted subcutaneously above the rat's limb. After 4 weeks, the device was removed, and the secretion of TGF-β1 was detected using ELISA (Supplementary Figure S6E), confirming long-term survival and secretion of GFs *in vivo*.

**FIGURE 4:**
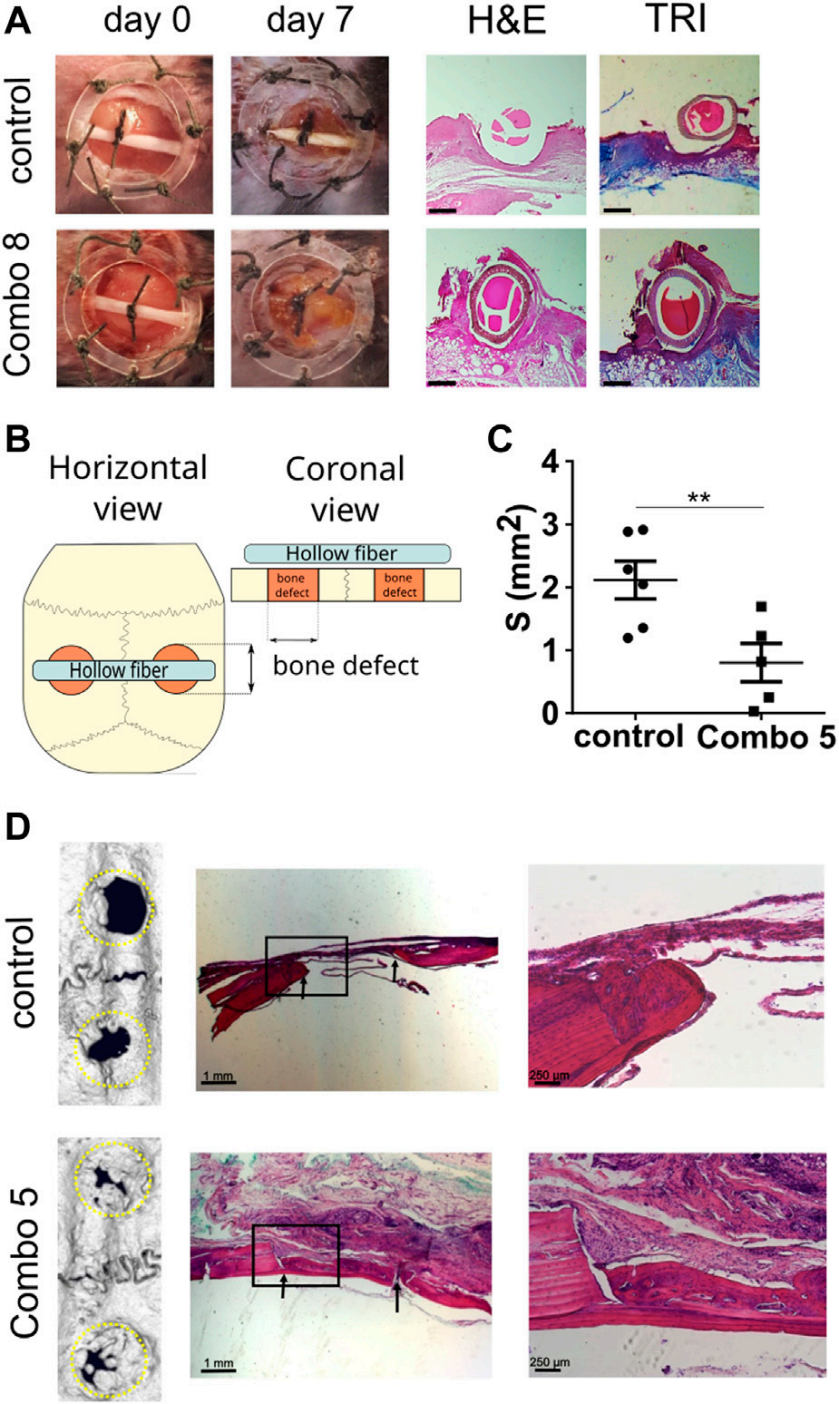
Cell-based device promotes excisional wound healing and bone regeneration in a calvarial defect. **(A)** Excisional wound was made in C57BL/6 mouse and fixed using a wound splint. The cell-based device with control cells (n = 4) or Combo 8 (n = 4) was inserted in the middle of the wound. After 7 days, healing was evaluated, and histological analysis was conducted. Representative photos of wounds at the beginning and after 7 days are shown. Histological analysis of wounds stained with H&E and Masson’s trichrome (TRI) is presented (scale bar represents 500 µm). **(B)** Schematic representation of the experimental setup. **(C)** Quantification of surface area measurements of defects based on micro-CT scans was performed. The average surface area per each animal is shown, and the mean ± SEM per condition are presented (n control = 6 animals, n Combo 5 = 5 animals), statistical analysis with a two-tailed *t*-test (*p < 0.05, **p < 0.01, ***p < 0.001). **(D)** Representative micro-CT scans of the calvarial defects after 2 months and histological analysis. Magnified insets are shown on the right. Arrows indicate the margins of the defect.

To evaluate the functionality and effectiveness of the cell device *in vivo*, we used a mouse excisional wound healing model. A silicone splint was placed around the wound to prevent wound contraction and to better recapitulate repair mechanisms underlying human wound healing (Wong et al., 2011). We prepared a cellular device secreting a combination of growth factors called Combo 8 (EGF, FGF-2, HGF, IGF-I, KGF, PDGF-B, TGF-β1, and VEGF-A; growth factors used are the same as in MIX, tested as abovementioned) where cells secreting a single GF were mixed at equal ratios. Cellular device Combo 8 was placed into the wound site and monitored for 1 week. On day 7, we observed that, in contrast to the control, a thin layer of newly formed soft tissue was covering the wound (Figure 4A; Supplementary Figure S6F), indicating the positive effect of the device on wound healing. No erythema, edema, and bleeding of tissue surrounding the device were observed, suggesting the inert properties of our device. A tissue analysis was carried out using conventional H&E and Masson’s trichrome staining. In animals treated with a combination of GFs, we detected increased epithelial gap closure and significant granulation tissue overgrowth. Granulation tissue, mainly composed of fibroblasts, collagen fibers, and angioblasts, stretched across the hollow fiber, which was absent in the wounds of control-treated animals (Figure 4A) that had the cellular device, carrying only control NIH-3T3 cells. In some tissue slides of GF-treated animals, newly formed arterioles were observed. Granulation tissue deposition extended from the base of wounds across the entire wound area. Mason’s trichrome staining showed increased *de novo* formation of collagen fibers in granulation tissue thus additionally confirming enhanced wound healing in GF-treated animals. GF serum levels of mice treated with Combo 8 did not differ from levels in sera of control device–treated animals, confirming that the GFs secreted from the device are restricted locally (Supplementary Figures S6G, I). The hollow fiber we are currently using is not biodegradable; however, the device can be surgically removed upon completion of treatment that leads to the complete resolution of the wound (Supplementary Figure S6J). We could therefore demonstrate that *in situ* local delivery of the eight growth factors potently enhanced the healing of cutaneous wounds in a mouse model.

### 3.3 Application of the cell device for bone regeneration

To demonstrate the adaptability of device preparation and its potential use for various applications, we decided to evaluate the device performance in the bone regeneration model. We used a rat calvarial defect model that is frequently used to observe bone regeneration, characterized by intramembranous ossification as a model of bone regeneration in the craniofacial region, which included dentistry (Spicer et al., 2012; van den Bos et al., 2008), where the cell-based device is placed over the calvarial defects (Figure 4B). First, the production of GFs and cell survival was tested. The cell device secreting only TGF-β1 was implanted over the defect and left for 4 weeks. Afterward, the device was removed and the secretion of TGF-β1 was evaluated using ELISA. As expected, the cell device continued to produce and secrete TGF-β1 confirming cell survival and the ability to produce growth factors for a prolonged period *in vivo* (Supplementary Figure S7A). For testing of therapeutic potential, a cellular device secreting IGF, FGF, PDGF, TGF-β, and VEGF (Combo 5) was chosen due to the previously reported effects of each of those growth factors (Devescovi et al., 2008; Krasnodembskaya et al., 2010; Park et al., 2018) and their abundance in platelet-rich plasma preparations (Devescovi et al., 2008). Defects were evaluated after 2 months using µCT, and the residual defect surface was quantified (Figure 4C). In the presence of a therapeutic device, new bone formation was significantly accelerated, resulting in the improved closure of defects (Figures 4C, D; Supplementary Figures S7B–E). After 8 weeks, bone defects of animals treated with a combination of growth factors were overgrown with a new bone. Detailed histological evaluation revealed that the regenerated bone had characteristics of an active bone remodeling process, with some areas still demonstrating characteristics of newly formed woven bone where collagen fibers were randomly oriented and more osteocyte lacunae were visible, which suggest higher synthetic activity. In addition, many cemental lines were formed because of calcium salt deposition on the bone surface before the process of regeneration and remodeling of woven bone to osteons. In most cases, there was a visible border or mineralization front between the lamellar bone and newly formed woven bone. More mature lamellar bone consisting of lamellae in which collagen fibers were oriented in a parallel manner, in contrast to newly formed woven bone, was consistently found on the cranial side facing the dura mater. On the skin-facing side of Combo 5–treated animals mature granulation tissue rich in connective tissue and vascular structures were evident. In the control group animals, no granulation tissue was noticed on the cranial or skin side of the defects. Bone regeneration was, however, confined to the defects’ edges and the area between the defect margins was occupied by connective tissue. Histological analysis revealed the formation of a growth cone in which centripetal bone tissue growth toward the center of the defect was evident. There was a distinguishable line of osteoblasts on the edge of newly formed bone that bordered the defect, showing osteogenic activity along the margins of the defect. Furthermore, we evaluated the survival of cells inside used cell devices. In both cases of the control and Combo 5 device, cell viability after 8 weeks (implanted *in vivo*) was around 70% (Supplementary Figure S7F). We measured the serum concentration of the selected growth factors and found no differences in systemic levels between Combo 5–treated animals and the controls (Supplementary Figures S7G–I), again confirming that the effect of the device remains restricted to the local environment, therefore decreasing any unwanted systemic proliferative effects. This confirms that elevated therapeutic concentrations of growth factors are not systemic but act at the site of the device, where they promote bone healing.

### 3.4 Spatiotemporal regulation of regeneration device based on inducible system

**FIGURE 5:**
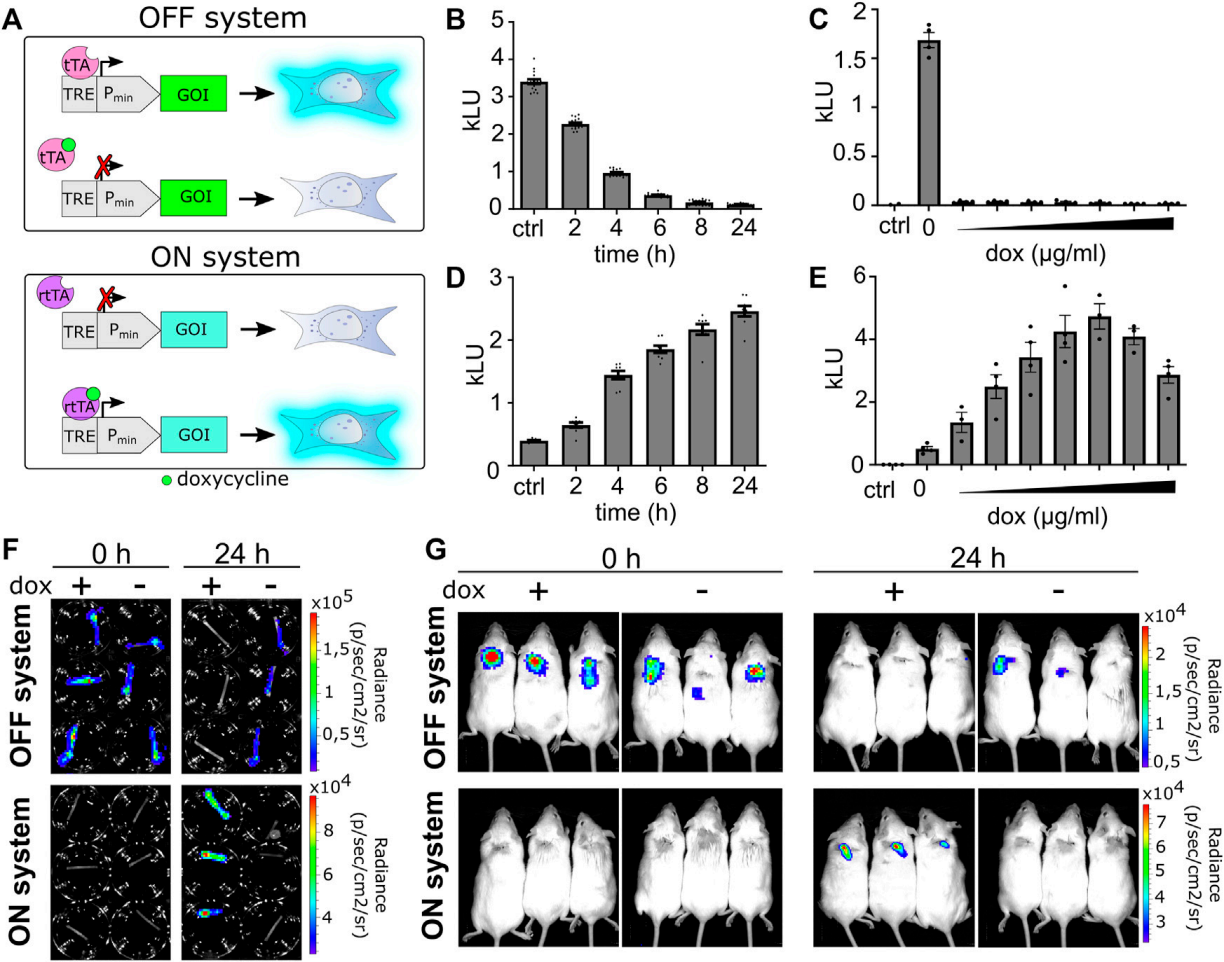
Validation of the ON–OFF genetic switch *in vitro* and *in vivo*. **(A)** Schematic representation of the OFF and ON systems. For an OFF system, in the absence of dox, the activator binds upstream of a minimal promoter and activates the gene of interest (GOI). Upon addition of doxycycline, the activator dissociates and the gene does not express. On the contrary, in the case of the ON system, gene is not expressed in the absence of an inducer. Upon induction with doxycycline, the activator binds to a minimal promoter and activates GOI. **(B)** Time-dependent deactivation or **(D)** activation of inducible systems. Data are presented as mean ± SEM (B, n = 16; D, n = 8), representative of three independent experiments. **(C,E)** Responsiveness of systems to doxycycline (0.01, 0.05, 0.1, 0.5, 1, 5, and 10 μg/mL). Data are presented as mean ± SEM (n = at least 3), representative of three independent experiments is shown. **(F)** Hollow fibers are filled with OFF or ON stable cell lines and photographed before induction with doxycycline (1 μg/mL; time 0) and after 24 h using an IVIS system. **(G)** Hollow fibers filled with OFF or ON stable cell lines are implanted subcutaneously into BALB/c mice and visualized at times 0 and 24 h after intraperitoneal doxycycline induction (20 μg/g) using the IVIS system.

Normal wound healing is a well-orchestrated process in which the release of various GFs and other molecules is fine-tuned to the different stages of wound healing. Spatiotemporal control over the secretion of growth factors is as important as the selection of growth factors (Koria, 2012). In contrast to the constant release of a single or set of growth factors, it would be advantageous if some could be secreted only in the early phase, others in the late phase, while the third group throughout the regeneration process. To better recapitulate the natural healing process, we introduced genetic switches that enable switching between two profiles of secreted factors during wound healing by an external chemical signal. Our system comprises two orthogonal transcriptional activators that bind to the Tet-binding sites (Tet-responsive element, TRE) but differ in binding in the presence of doxycycline (dox). The ON system comprises Tet-On 3G transactivator protein (3G) that upon induction with doxycycline binds to TRE (TRE3GS) and activates the gene of interest but cannot bind to TRE in the absence of doxycycline (Figure 5A). For the OFF system, a doxycycline-controlled transactivator (tTA) was prepared by fusing TetR with the VPR activation domain (Chavez et al., 2016). In the absence of doxycycline, tTA binds to TRE and activates gene expression, while the addition of doxycycline prevents its binding and consequently represses the activation (Figure 5A).

First, we tested the responsiveness of the systems using a luciferase reporter. Time-dependent and doxycycline concentration–dependent activation of the luciferase ON system (LucON) or decrease of activity in the luciferase OFF system (LucOFF) was confirmed (Figures 5B–E). Based on the results (Figures 5C, E), 1 μg/mL doxycycline was selected as the switching signal for further *in vitro* experiments. To determine if cells respond to doxycycline stimulation when encapsulated, the hollow fiber was filled with cells stably transfected with the LucON or LucOFF system. As expected, encapsulated cells containing LucON expressed luciferase upon doxycycline stimulation, while luciferase expression in cells containing LucOFF decreased (Figure 5F, S8 A). Next, we investigated the responsiveness of a cell device *in vivo*. Cells expressing either LucON or LucOFF were encapsulated into a hollow fiber, and the device was implanted into mice. Luciferase activity was measured before and 24 h after dox administration. A significant increase in the signal was observed in the case of LucON and a decrease in signal in the case of the LucOFF system (Figure 5G, S8 B), confirming functional switching of a cell device *in vivo*.

**FIGURE 6:**
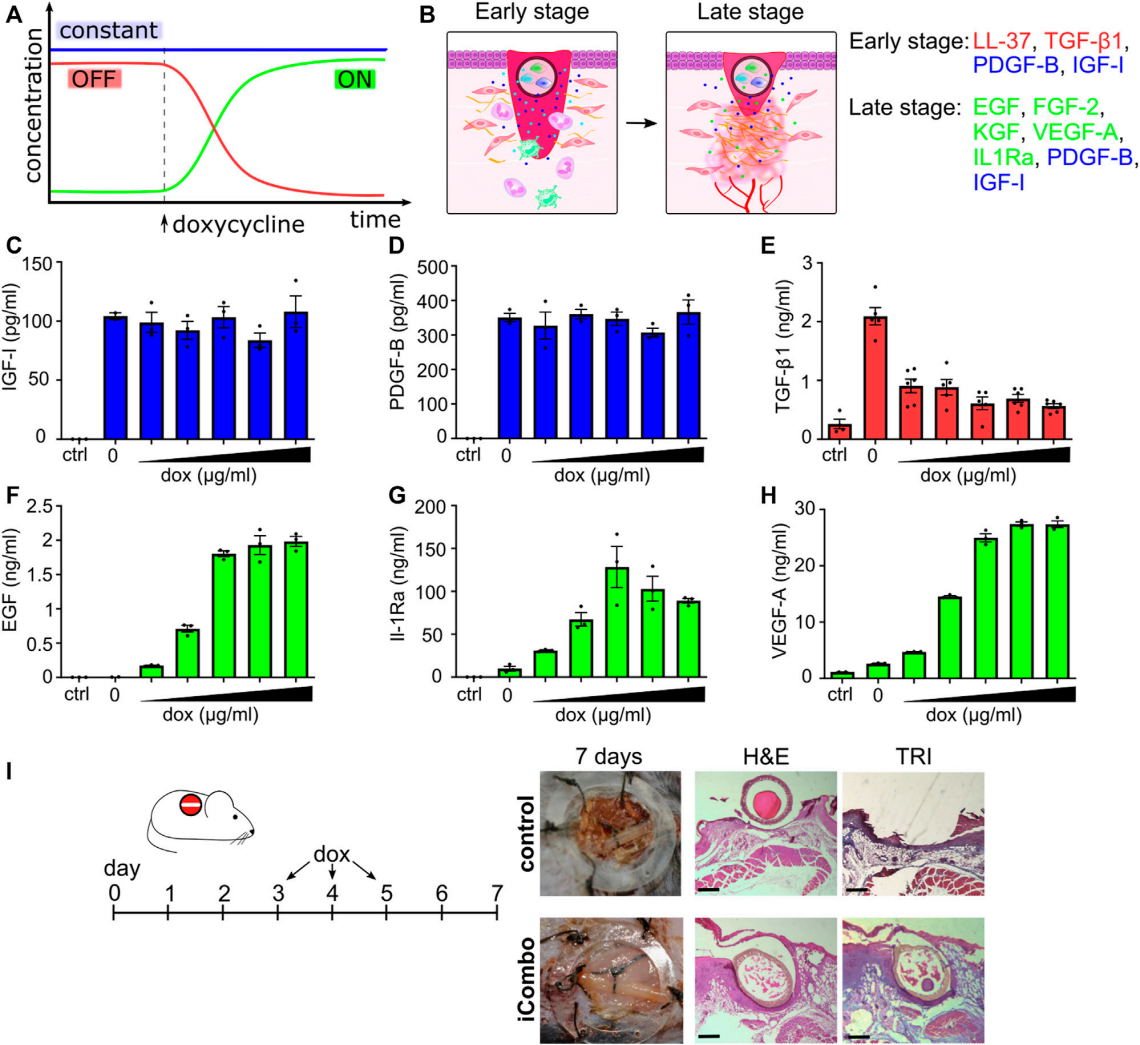
Regulated temporal release of trophic factors promotes wound healing. **(A,B)** Schematic representation of the inducible cell system design. IGF and PDGF are stably expressed through therapy. In the first phase, LL-37 and TGF-β1 are expressed under the control of the OFF system until the addition of doxycycline; when their production is decreased and the device switches ON, producing EGF, FGF-2, IL1Ra, KGF, and VEGF, in addition, to stably expressed GF. **(C–H)** Responsiveness of selected stable cell lines on stimulation with different concentrations of doxycycline (0.01, 0.05, 0.1, 0.5, 1, and 5 μg/mL). Stable cell lines, each expressing a single factor, were seeded into a 96-well plate, and doxycycline was added. Concentrations of GFs are evaluated after 24 h using ELISA. The mean ± SEM of **(C,D,F–H)** n = 3 and **(E)** n = at least 4 are shown. **(I)** *In vivo* testing of the iCombo device. The inducible device was tested *in vivo* on an excisional wound model on C57BL/6 mice. On days 3, 4, and 5, doxycycline (20 μg/g) was administered intraperitoneally. On day 7, wound healing was evaluated and sampled for histological analysis and stained with H&E (left) and trichrome (right) (scale bar represents 500 µm).

After confirming the functionality of the switch on a reporter system, we tested the implementation of the system to regulate the secretion of GFs playing an active role in regeneration. Healing comprises different stages, each characterized by the presence of a distinctive set of GFs and other molecules. For that reason, we prepared a therapeutic device comprising of a mixture of different stable cell lines constitutively producing or induced to produce selected bioactive molecules that in the early stages of therapy augment the infiltration of immune cells and release antimicrobial peptide LL-37, facilitating the transition into the next phase of healing. In the late phases of healing, the therapeutic device should stimulate keratinocyte, fibroblast, and endothelial cell migration to improve healing. In the selected setup, the device produced PDGF and IGF-I constantly throughout all stages of therapy (Figures 6A, B). The reason for this is that PDGF has an important role in wound healing and is released in large amounts upon injury (Barrientos et al., 2008). IGF-I is also important in the normal wound-healing process, while its levels are decreased in chronic wounds (Werner and Grose, 2003). In the first stage of therapy, in addition to PDGF and IGF, TGF-β1 and antimicrobial peptide LL-37 are expressed. Both are expressed under natural wound healing and are released shortly after the injury, and have a positive effect on healing (Duplantier and van Hoek, 2013; Grönberg et al., 2014). In addition to its antimicrobial effects, LL-37 enhances re-epithelialization and granulation tissue formation (Carretero et al., 2008) and modulates GFs expression (Adase et al., 2017). TGF-β1 has a major role in wound healing affecting inflammation, proliferation of fibroblasts, angiogenesis, and matrix remodeling (Penn et al., 2012) but is not desired in the later phases of wound healing since it can lead to excessive scarring (Grönberg et al., 2014), therefore its production should be turned OFF at the later stage. Upon induction with doxycycline, the device switches to the second stage and produces EGF, FGF-2, KGF, VEGF-A, and IL-1Ra (Figures 6A, B). EGF, FGF-7 (KGF), and FGF-2 play important roles in re-epithelization.

All constructs and cell lines responded as expected upon induction with doxycycline. The addition of dox did not influence the production of IGF-I and PDGF-B (Figures 6C, D). In the case of TGF-β1 and LL-37 under the control of the OFF system (Figure 6E, S8 C), production decreased upon doxycycline induction, and in the case of EGF, IL-1Ra and VEGF-A, under the ON system (Figures 6F–H), the concentration of these proteins increased upon induction with doxycycline.

To test the functionality of an inducible cell device *in vivo*, the iCombo9 device was implanted into skin wounds in a mouse model. In the first stage (early stage; wound clearing/cell migration), the device produced and secreted PDGF-B, IGF-I, TGF-β1, and LL-37. After 3 days, doxycycline was administered to the mice (days 3–5) which caused the device to switch to the production of EGF, FGF-2, KGF, VEGF-A, and IL-1Ra (in addition to PDGF-B, IGF-I, which were secreted constitutively) promoting the second stage (late phase; granulation, angiogenesis, and wound contraction). The device resulted in *de novo* tissue formation, covering the hollow fiber in clear contrast to the control animals (Figure 6I; Supplementary Figure S9). The histology analysis of the wounds after 7 days (H&E and Mason’s trichrome staining) confirmed improved wound healing based on accelerated epithelial gap closure and granulation tissue with collagen fiber overgrowth across the device. The results thus demonstrated that an inducible device with the incorporated switch in growth factor expression acts favorably on wound healing.

## 4 Discussion

Inefficient healing and regeneration present multifaceted hurdles for regenerative medicine. Single growth factors have been extensively studied in therapeutic applications and clinical trials yet provided limited efficacy or even negative adverse effects (Eming et al., 2014). Several studies have also observed an improved effect of a combination of two or three growth factors (Martino et al., 2014). In this study, we built a library of cell lines that were engineered to secrete a selected growth factor. Such cells can in principle be combined at will to provide the best therapeutic combination of growth factors for each selected application. Stem cells have been reported to contribute to regeneration mainly by secretion of multiple growth factors. In this study, we imitated stem cells by enabling the simultaneous secretion of multiple growth factors by using designed cells. In accordance with previous findings, SC-conditioned medium accelerated cell migration, but a combination of eight growth factors secreted by a combination of engineered cells was even more efficient. The secretome analysis of cell combination designed to secrete the eight growth factors revealed enrichment of proteins involved in proliferation, cytokine production, immune cells chemotaxis, blood tissue development, epithelial–mesenchymal transition (EMT), growth factors signaling, and several other processes that influenced wound healing, providing additional insight into the mechanisms of cell-based device action in regeneration. RNA analysis revealed that the combination of growth factors in treated fibroblasts enhances pathways that lead to collagen and extracellular matrix formation.

Encapsulation of the selected cell combinations confined into a semipermeable container enabled the continuous *de novo* synthesis of target proteins coupled with beneficial safety properties and retrievability after the termination of therapy. Although (PVDF) fiber was used for encapsulation, any other biocompatible or even biodegradable material could be used (Bochenek et al., 2018). Encapsulated cells have already been used to support myocardial regeneration (Yu et al., 2010; Park and Yoon, 2018), bone regeneration (Moshaverinia et al., 2014), and the treatment of neurodegenerative diseases (Emerich et al., 2013). We demonstrated that GF-secreting stable cell lines endure encapsulation *in vitro* and *in vivo* and, as expected, produce and secrete desired soluble molecules. The cell device secreting multiple (eight) growth factors improved healing of incisional cutaneous wound in an animal model. A different cell device can be easily prepared in a mix-and-match manner by selecting the desired combination of growth factors/cells in a predetermined cell ratio. For demonstration, a different set of growth factors suitable for bone regeneration was selected resulting in an improved closure of calvarial defects in comparison to the control. Collectively, these data confirm the functionality of the cell-based device *in vivo* and also show that the device is easily adaptable for various regenerative applications. Furthermore, the serum levels of secreted factors were not increased, suggesting predominately local action, thus adverse side effects due to increased systemic levels were neither expected nor observed. Innovative macro encapsulation containers might further prolong and increase the performance of engineered devices (Bose et al., 2020) and facilitate device retrieval.

In the process of normal wound healing, different growth factors are present at different stages of healing, with some even negatively affecting a certain phase of regeneration. TGF-β1 has been shown to accelerate wound healing in early phases but can lead to scarring in the later phases of healing (Liu et al., 2016). To overcome this, an inducible cellular device was introduced that changes the profile of secreted factors. The tested device uses a small molecule approved for human use to switch the secreted GF profile, with three different regimens for groups of associated GFs (ON/ON, ON/OFF, and OFF/ON). Other solutions could also be envisioned, e.g., a bistable genetic switch that can be switched to a desired stable state using a small chemical inducer (Lebar et al., 2014), which could also be replaced with light-responsive switches (Strickland et al., 2012; Muller et al., 2014). An additional improvement could be based on the detection of an endogenous signal characteristic for the end of an inflammatory wound healing phase coupled to a circuit that could enable the appropriate response (Ye et al., 2016; Smole et al., 2017; Chassin et al., 2017). This would enable an autonomous change of the therapeutic regime to achieve the best effect at the appropriate moment.

In this proof-of-concept study, we designed cell devices that mimic and supersede the positive effects of stem cell therapy. This improves the regeneration processes with the potential for various therapeutic indications. Encapsulation of cells and retrieval of the device ensured additional safety, and the introduction of inducible elements provided better control over the set and timing of the secretion of soluble factors. Due to their ease of preparation and tight control over the selection of therapeutic molecules, the cellular device represents a promising alternative to existing therapies and could be used in other medical applications that require the application of therapeutic proteins for an extended period.

## Data Availability

The data sets presented in this study can be found in online repositories. The names of the repository/repositories and accession number(s) can be found in https://data.mendeley.com/datasets/pwtp5nc6m3/1, doi:10.17632/pwtp5nc6m3.1.
