# Supplementary material for: Engineered combinatorial cell device for wound healing and bone regeneration
Source: Front Bioeng Biotechnol. 2023 May 10;11:1168330. doi: 10.3389/fbioe.2023.1168330 (PMC10206319; doi:10.3389/fbioe.2023.1168330)
Supplement: Supplementary file 1 [file DataSheet1.PDF]

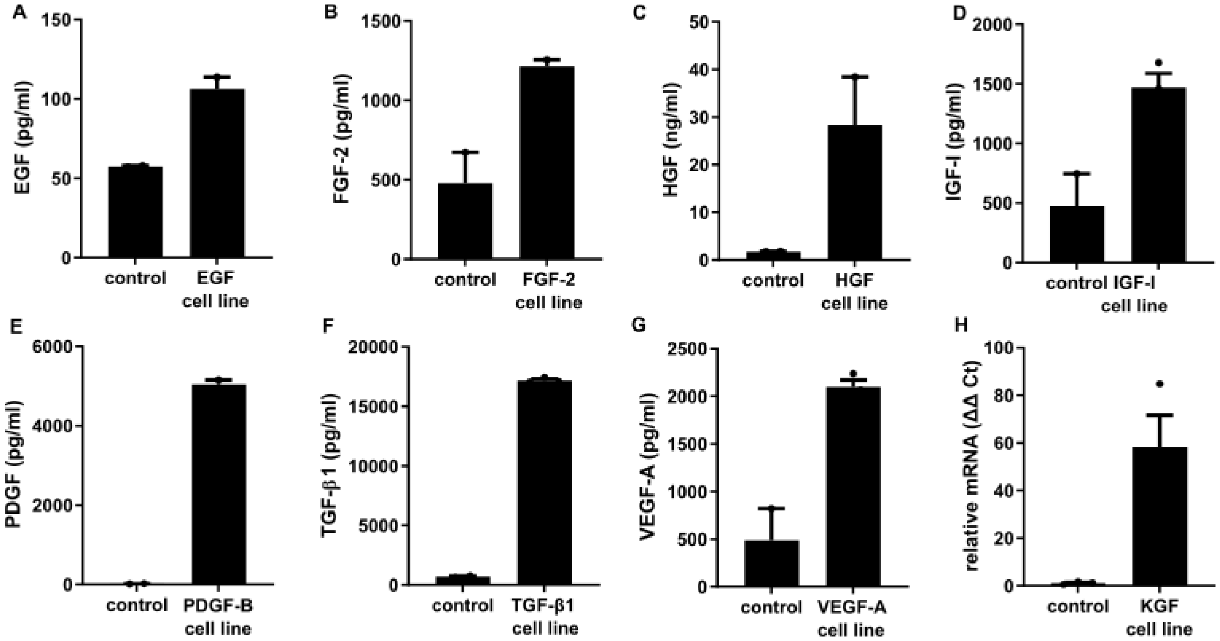

Supplemental Figure 1: Expression of growth factors by stable cell lines. A – G) Supernatants of stable cell lines were collected and the concentration of growth factors was measured using ELISA. Untransfected NIH3T3 cell supernatants were used as control. H) mRNA of stable cell lines expressing KGF was isolated and expression of KGF was confirmed using qPCR. mRNA of untransfected NIH3T3 cells was used as a control.

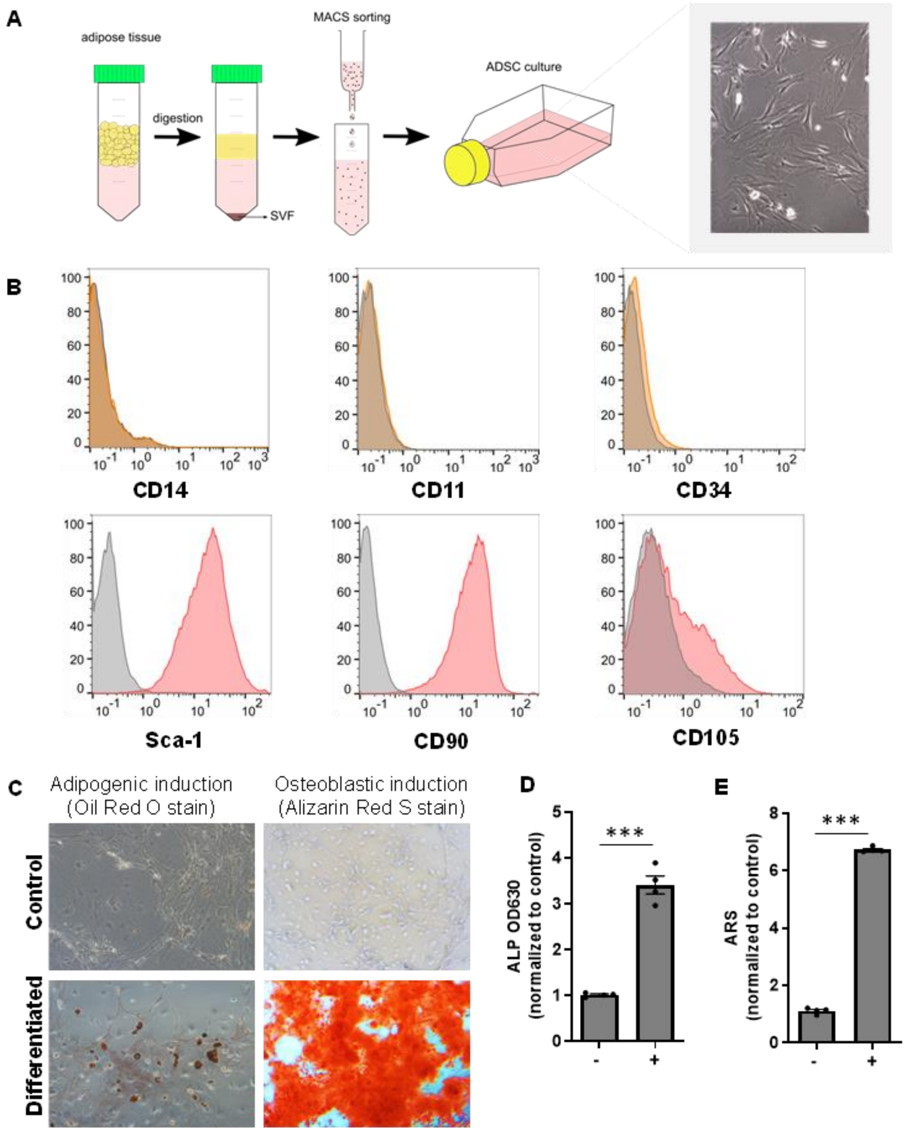

Supplemental Figure 2: Isolation and characterization of mouse adipose-derived stem cells. A) Stem cells were isolated from adipose tissue of adult C57BL/6 mice. Isolated cells were adherent spindle-shaped. B) Isolated cells were characterized using flow cytometry. They were negative for surface markers CD14, CD11, CD34 and positive for surface markers Sca-1, CD90 and CD105. Histograms are representative of two independent replicates. C) Differentiation potential of isolated stem cells was confirmed with differentiation into adipocytes and osteoblasts. Differentiated cells were stained with Oil Red O (adipocytes) or Alizarin Red S (osteoblasts). Representative images of 3 independent experiments are shown. D) Presence of alkaline phosphatase (ALP) in control (-) and osteoblast differentiation induced (+) cells was determined using Quanti-Blue dye and measuring absorbance at 630 nm. Data are shown as mean ± SEM (n = 4), representative of two independent experiments is shown. E) Alizarin Red S (ARS) dye was extracted from control (-) and osteoblast differentiation induced (+) cells and quantified via measurement of absorbance at 405 nm. Data are shown as mean ± SEM (n = 4). Statistical analysis with a two-tailed t-test (*p < 0.05, **p < 0.01, ***p < 0.001).

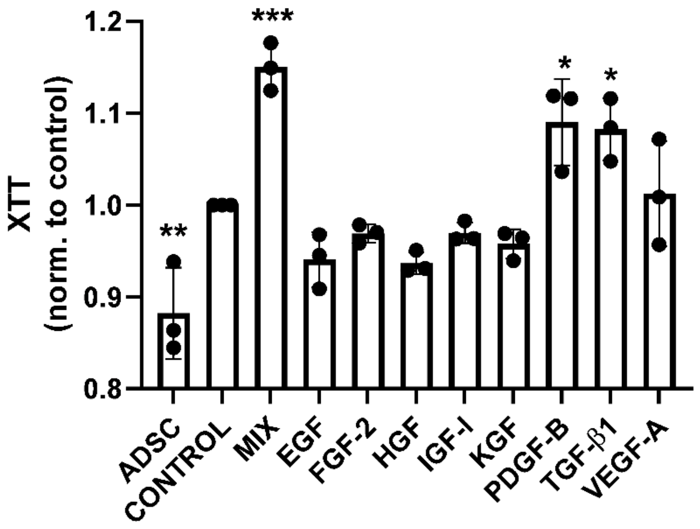

Supplemental Figure 3: Effect of conditioned media on MSF fibroblast metabolic activity. MSF cells were treated with conditioned media for 24 h. XTT reagent was added to cells and metabolic activity was measured. The mean and SD of the average values of three independent experiments are shown. Statistical analysis with one-way ANOVA compared to control (*p <0.05; **p < 0.01; *** p < 0.001).

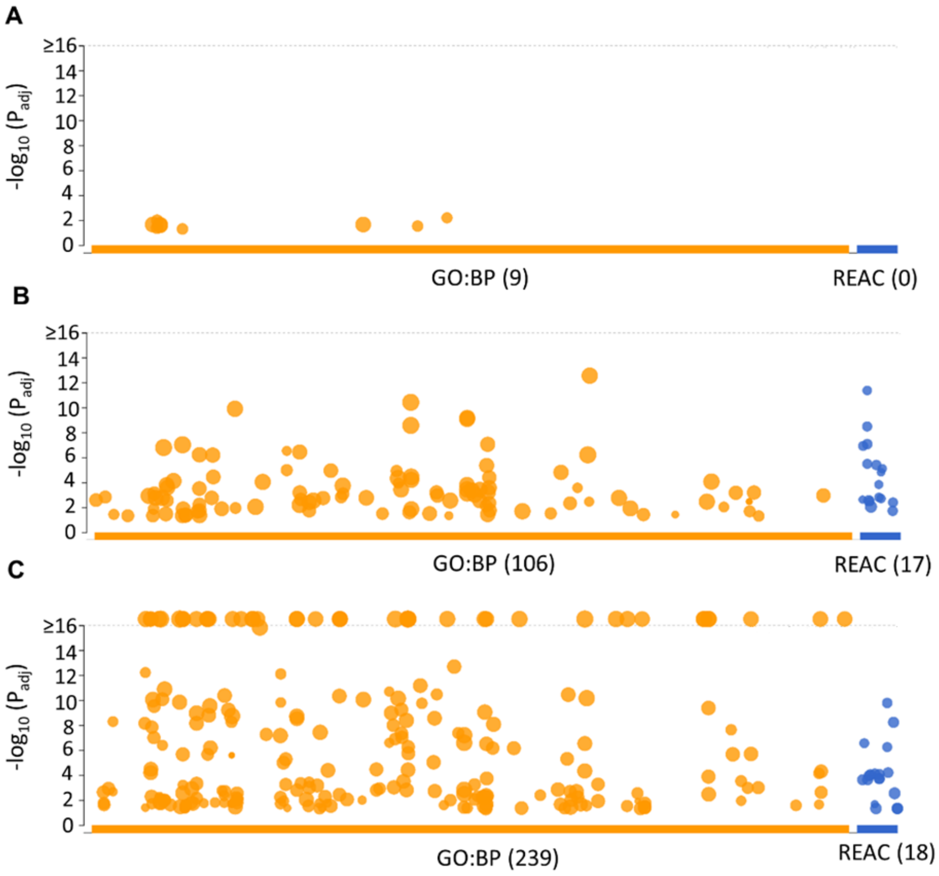

Supplemental Figure 4: Gene set Enrichment analysis of differently expressed genes in MSF cells. A) Enriched pathways in downregulated genes of SC-treated cells compared to control. B) Enriched pathways in upregulated and C) downregulated genes in MIX treated cells compared to control.

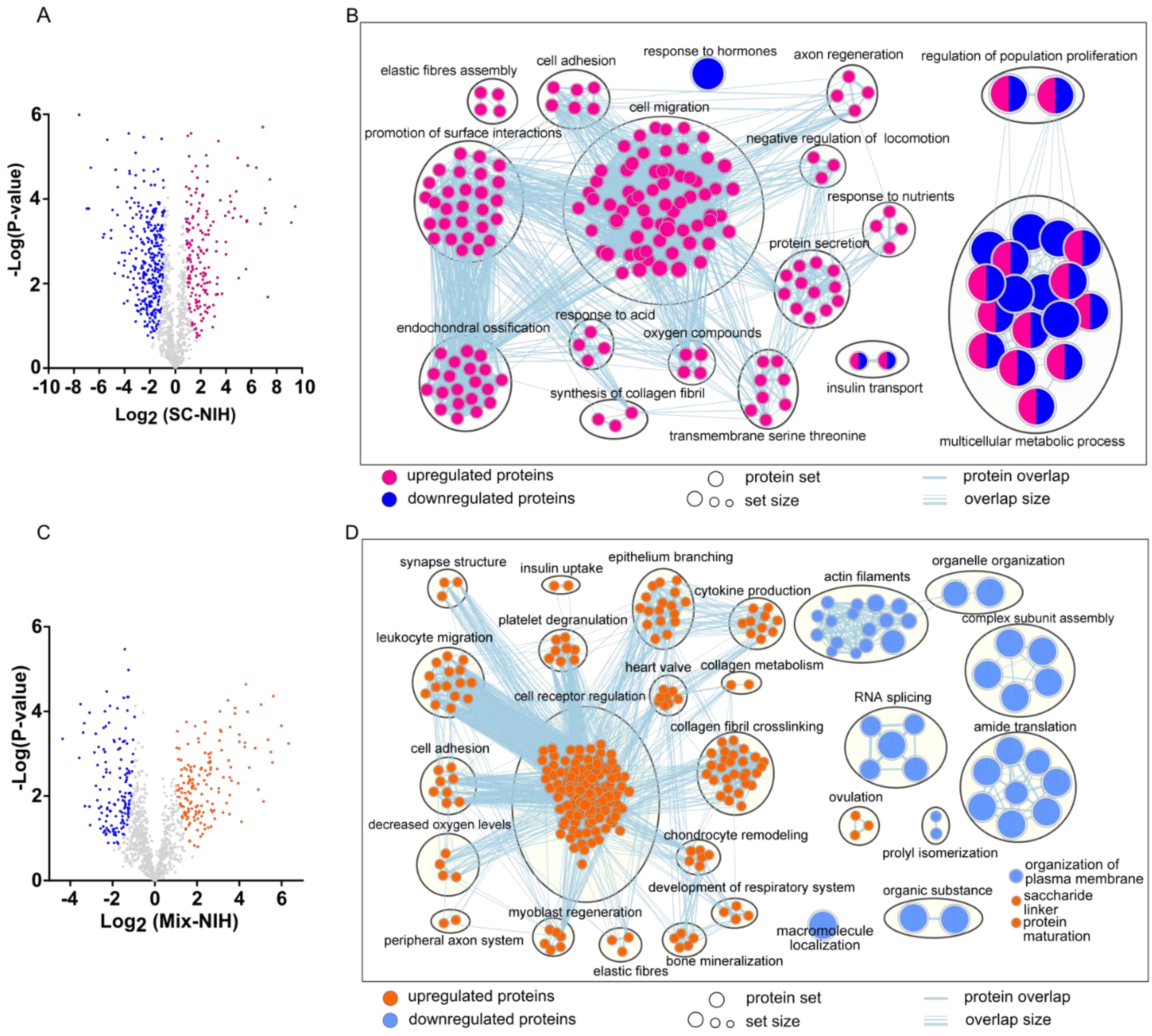

Supplemental Figure 5: Protein abundance in media conditioned with SC or MIX compared to control. A) Protein abundance in SC conditioned media compared to control. B) Enrichment analysis of up- and downregulated proteins in SC media compared to control. C) Protein abundance in MIX conditioned media compared to control. D) Enrichment analysis of up- and downregulated proteins in MIX media compared to control.

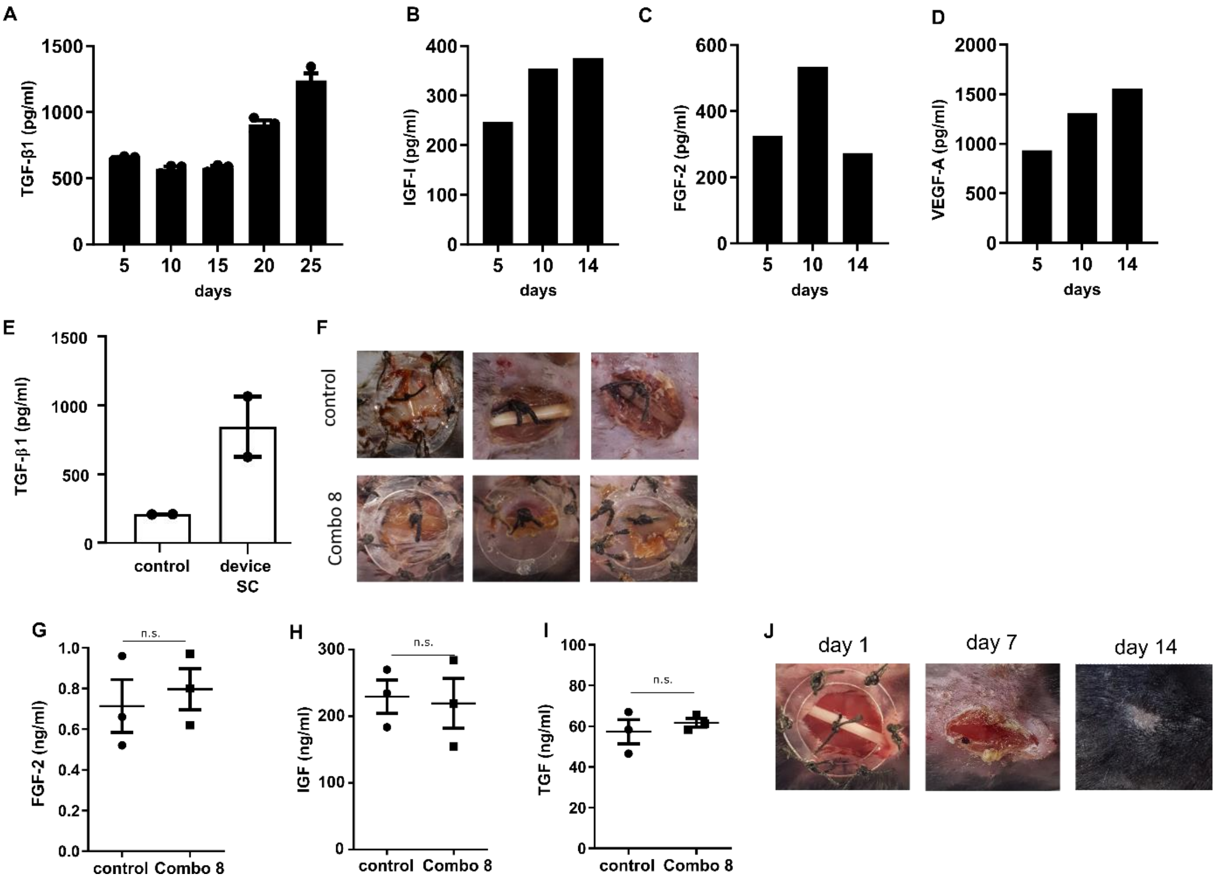

Supplemental Figure 6: Design and validation of cellular device. A-D) To confirm long-term production and secretion of GF (TGFβ1, IGF-I, FGF-2 and VEGF-A) from the cell device, the device was cultured 14 - 25 days. Media was changed daily and supernatants were analyzed using ELISA. Secretion of GF from the cell device was confirmed using supernatants. E) Device secreting TGF-β1 and control device (untransfected NIH3T3 cells) were implanted subcutaneously (sc). After 4 weeks device was removed and secretion of GF was evaluated using ELISA. For control and sc implantation, 2 animals per group were used.* F) Wound healing after 7 days of treatment with Combo8 or a control device. Only wounds (animals) that are not shown in the main figure are presented here. G – I) Serum concentration of growth factors from 3 animals with implanted control or Combo8 cell device after 7 days measured using ELISA. Statistical analysis with a two-tailed t-test (n.s. = non-significant). J) Surgical removal of Combo8 cell device after 7 days leads to complete healing. * control in S6E is the same as in S7A.

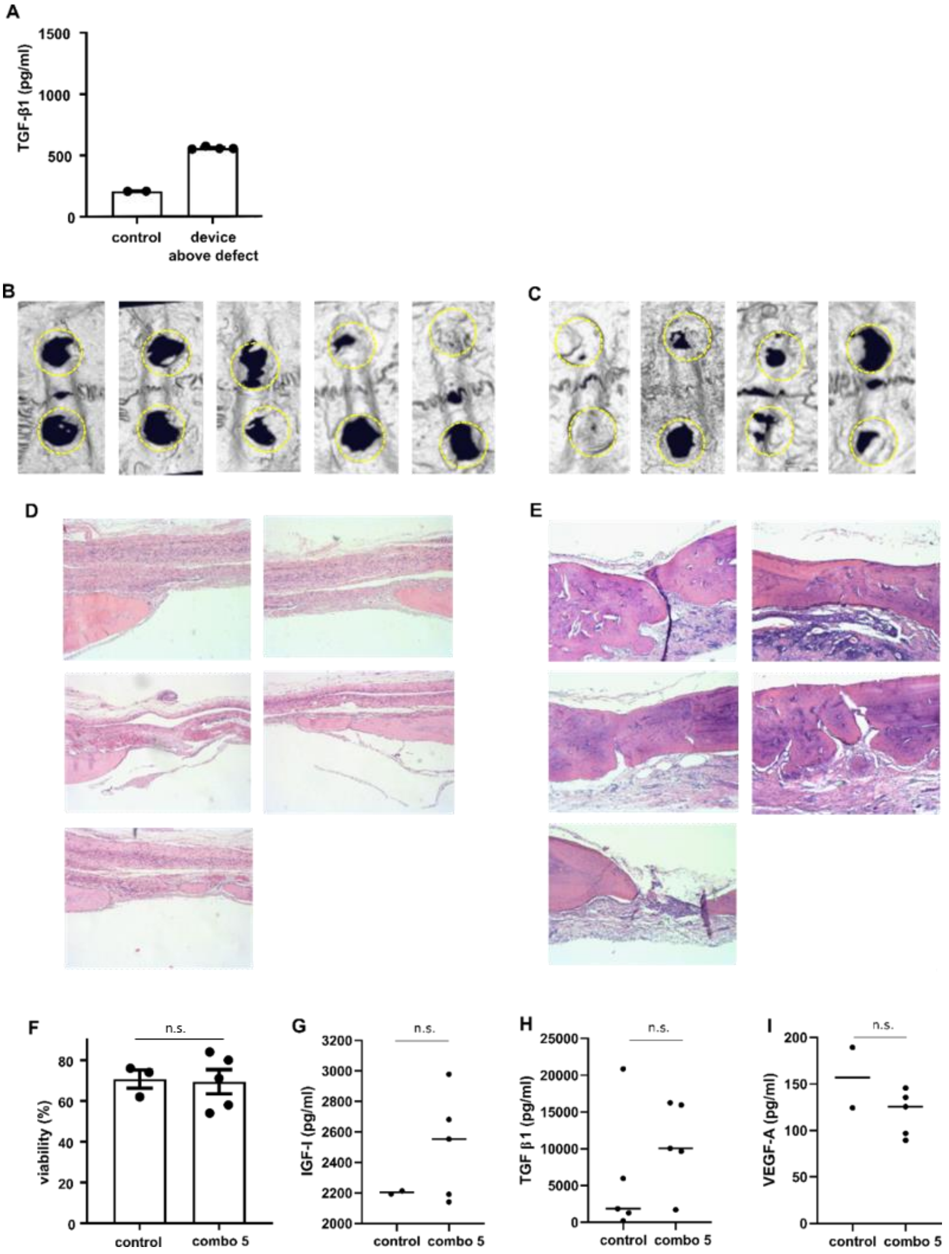

Supplemental Figure 7: Healing of calvarial defects. A) Cell viability after removal of cell device from calvarial defect. Device secreting TGF-β1 and control device (NIH3T3 cells) were implanted over the bone defect. After 4 weeks the device was removed and secretion of GF was evaluated using ELISA. For control 2 animals per group were used; for bone defect 4 animals were used.* B) μCT scans of calvarial defect with implanted control or C) Combo 5 cell device after 2 months. D) Histological analysis of control or E) Combo5 cell device. F) The survival of cells inside the used cell device. Statistical analysis with a two-tailed t-test (n.s. = non-significant). G-I) Serum concentration of growth factors from animals with implanted control or Combo5 cell device (each dot represents a single animal). In the case of control animal sera, a limited volume of samples allowed only the measurement of selected growth factors. Statistical analysis with a two-tailed t-test (n.s. = non-significant). * control in S7A is the same as in S6E.

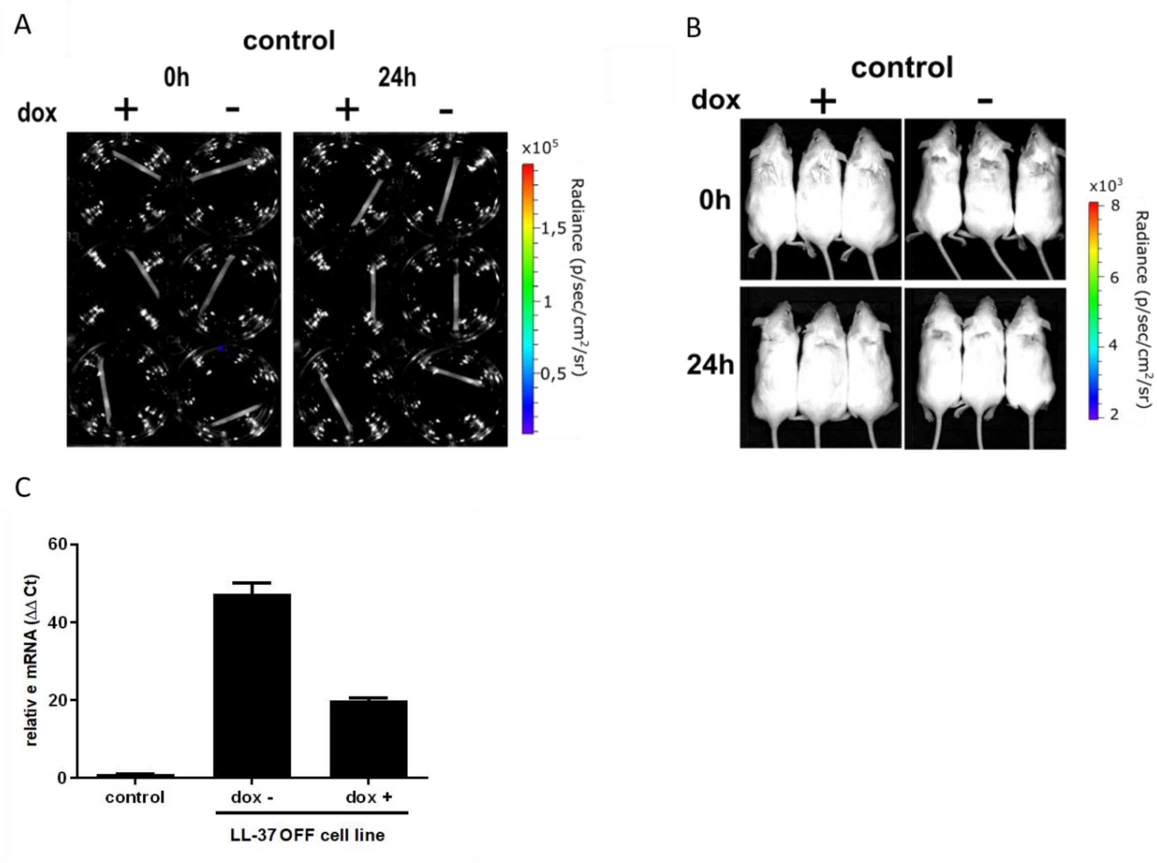

Supplemental Figure 8: Validation of genetic switch *in vitro* and *in vivo* and validation of inducible expression of LL-37 A) Nontransfected cells NIH3T3 were encapsulated in hollow fiber and visualized with IVIS system at time 0 and 24 h after dox induction. B) Hollow fibers with nontransfected NIH3T3 cells were implanted subcutaneously into mice and visualized using the IVIS system at times 0 and 24 h after dox induction. C) Expression of LL-37 OFF in the presence or absence of dox (1µg/ml).

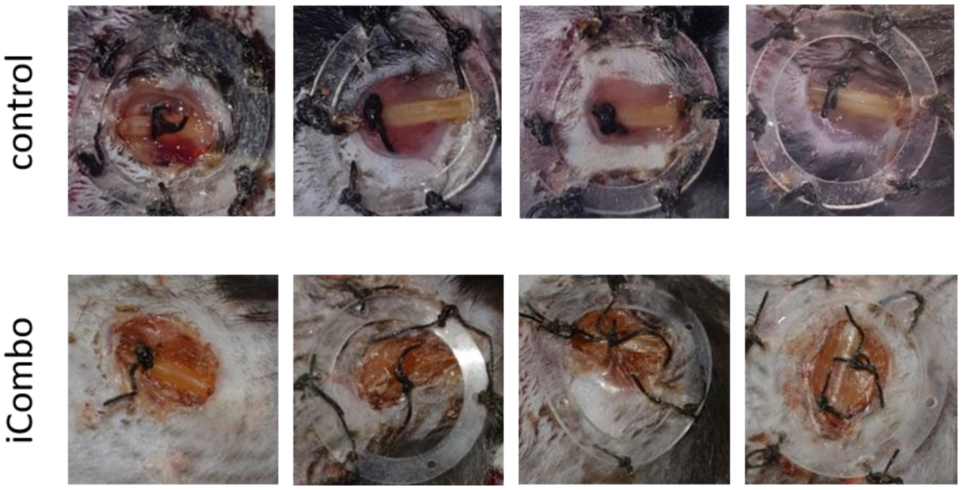

Supplemental Figure 9: Wound healing in mice treated with iCombo9 and control device. Wounds of animals after 7 days not presented in Figure 6 are shown.
